# Acknowledgement to Reviewers of *Sensors* in 2015

**DOI:** 10.3390/s16010136

**Published:** 2016-01-21

**Authors:** Sensors Editorial Office

**Affiliations:** MDPI AG, Klybeckstrasse 64, CH-4057 Basel, Switzerland; sensors@mdpi.com

The editors of *Sensors* would like to express their sincere gratitude to the following reviewers for assessing manuscripts in 2015. 

We greatly appreciate the contribution of expert reviewers, which is crucial to the journal’s editorial decision-making process. Several steps have been taken in 2015 to thank and acknowledge reviewers. Good, timely reviews are rewarded with a discount off their next MDPI publication. By creating an account on the submission system, reviewers can access details of their past reviews, see the comments of other reviewers, and download a letter of acknowledgement for their records. In addition, MDPI has launched a collaboration with Publons, a website that seeks to publicly acknowledge reviewers on a per journal basis. This is all done, of course, within the constraints of reviewer confidentiality. Feedback from reviewers shows that most see their task as a voluntary and mostly unseen work in service to the scientific community. We are grateful to our reviewers for the contribution they make.

A. Fimbres-Weihs, GustavoHu, JiankunPizzo, Silvio DelAanstoos, James V.Hu, FeiPlaninc, RainerAbawajy, JemalHu, XiaogongPlant, Richard E.Abbaszadeh, S.Hu, ZhichaoPlasqui, GuyAbbod, Maysam F.Hu, Jwu-shengPochiraju, Kishore V.Abdel-Motaleb, Ibrahim M.Hu, XiaopingPoertner, RalfAbdel-Wahab, Wael M.Hu, Zhi-QiangPoisel, HansAbdelkefi, AbdessattarHu, HongxinPoletkin, KirillAbdeslam, Djaffar OuldHu, ChengchenPolidori, Maria CristinaAbdi, HamidHua, Hui GuoPomalaza-Ráez, CarlosAbdul-Aziz, AliHua, Kai-LungPomares Hernandez, Saul E.Abdulhalim, IbrahimHuang, QinghuaPombar, MiguelAbot, Jandro LHuang, Tien-ChiPonchak, George E.Abrudan, Traian E.Huang, Yueh-MinPopovics, John S.Abtahi, FarhadHuang, Yo-pingPoppe, LaszloAbu-Nabah, BassamHuang, XiaopengPortalés, CristinaAbuzneid , Abdel-ShakourHuang, Qing-AnPortilla, Jorge HigueraAcharya, Rajendra UdyavaraHuang, Ming-ChunPoslad, StefanAchir, NadjibHuang, JianxiPosner, JonathanAdamatzky, AndyHuang, YiPostalache, OctavianAdamo, MariaHuang, WenbinPotkay, JosephAdams, MichaelHuang, Guang BinPotter, MarkAddabbo, TommasoHuang, YingPotter, LeeAderohunmu, FemiHuang, YanboPoulymenopoulou, MikaelaAernouts, BenHuang, WeiminPradell, LLuisAeron, ShuchinHuang, ChengzhiPrades, Joan Daniel Afseth, NilsHuang, Jui-chenPrager, JensAfsharipour, BabakHuang, Jan-ChanPrakash, RanganathanAgam, NuritHuang, ZhiyiPrasath, SuryaAggelis, DimitriHuang, HaifengPrat, MariaAggelis, Dimitrios G.Huang, Mong-HanPreatoni, DamianoAgili, Sedig S.Huang, Long-sunPRECUP, Radu-EmilAgrafiotis, PanagiotisHuang, Yuan-KoPreethichandra, PreethiAgrawal, DharmaHuang, LeiPremebida, CristianoAgrawala, Ashok K.Huang, WeiPreuveneers, DavyAgudo, J. EnriqueHuang, Chih-ChingPrida, V. M.Agüera, FranciscoHuang, Xing-JiuPrieto, JavierAguilar, Juan JoséHuang, XiaoleiPrins, FerryAguilar, MónicaHuang, ChongProctor, ChrisAguilera, CristhiaHuang, ZhiyaoPropastin, PavelAguirre-Salado, Carlos A.Huang, JiePrudenzano, FrancescoAhmad, ZeeshanHuang, Jiung-yaoPruski, AlainAhmadabadian, Ali HosseininavehHuang, YikunPryss, RüdigerAhmed, MahmoudHuang, Genin GaryPsimoulis, PanagiotisAhmed, SamirHuang, Shih-ChiaPu, FangAhn, NamsuHuang, Chih-yungPu, LinaAiello, OrazioHübers, Heinz-wilhelmPu, ShengliAjib, WHuberty, BrianPudlo, PhilippeAjisafe, ToyinHuebner, ChristofPuig, VicencAKAI, DaisukeHueni, AndyPulsford, RichardAkbar, SheikhHugentobler, UrsPunchihewa, KasunAkhloufi, Moulay A.Hughes, DannyPurser, AutunAkhtari, MassoudHull, Maury L.Püschel, Gerhard P.Akiba, YasutadaHullo, Jean-FrançoisPutrino, DavidAkitsu, TetsuyaHung, Jui-chungPyeon, CheolhoAksakal, Sultan KocamanHung, PatrickPylatiuk, ChristianAl-Anbagi, Irfan S.Hung, H.l.Pyun, Jae-chulAl-Anbuky, AdnanHunsche, MauricioQi, FengAl-Ani, AhmedHuntsberger, TerryQian, Cheryl ZhenyuAl-Hamadi, AyoubHuo, XingliangQian, YouAl-Mukhtar, M.Huotari, MattiQiao, LiAl-Sarawi, Said F.Hur, ShinQin, FeiAl-Shabi, MohammadHur, JinQin, PeiyuanAl-Yasiri, AdilHurley, William GerardQin, YuanweiAlaman, XavierHussein, MohamedQiu, YingAlander, JarmoHussein, Walid B.Qiu, TieAlanis, ArnulfoHuthwaite, PeterQiu, LiangAlbert, AmosHütt, ChristophQiu, WeibaoAlcaraz, Juan J.Hutter, TanyaQu, PengAlcarria, RamonHuwald, HQu, FengzhongAlchanatis, VictorHwang, YoolaQu, GangAleixandre, ManuelHwang, Dong-HwanQu, YonghuaAlenyà, GuillemHwang, HoyoungQuaglia, DavideAlessandrini, AndreaHwang, Wen-jyiQuagliotti, FulviaAlexeev, AlexanderHwang, Jeng-NengQureshi, AnjumAlfredson, HenrikHwang, YonghaRaahemifar, KaamranAlgar, RussHwang, Jenq-NengRablau, Corneliu I.Ali, MubarakHwang, Won-JooRadaelli, LauraAli, Muhammad RazaHwang, Han-JeongRadamson, Henry HAli, Syed TahaHwang, Min-shiangRadecki, JerzyAli Tavallaei, MohammadHwang, GilguengRaghavan, SeethaAlippi, AdrianoHwang, Chi-HungRagulskis, MinvydasAlmeida, AitorIadicicco, AgostinoRahaman, DMAlmeida, Catarina R.Ibaraki, SoichiRahman, Md. ArafaturAlmeida, RuteIckowicz, Adrien A.Rahman, ShahedurAlmond, Darryl P.Iera, AntonioRahmani, RahimAlonso, Jesús B.Ieropoulos, IoannisRahnejat, HomerAlonso-Garcia, SergioIervolino, ElinaRaitoharju, MattiAlonzo, MichaelIglesias, José AntonioRakić, Aleksandar D.Altdorff, DanielIgnjatovic, AleksandarRamadan, QasemAlthunibat, SaudIgual, RaulRamanandan, A.Alvarez, JesusIgual, Francisco D.Ramanavicius, ArunasAlvarez, Jose M.Ihalainen, PetriRamírez Muñoz, DiegoAlvarez, Juan CarlosIkeda, TetsushiRamisa, ArnauAlves, MárioIkonen, ErkkiRamoelo, Abel (selaelo)Amditis, AngelosIkonomidou, Vasiliki N.Ramos, Alexandre Carlos BrandãoAmft, OliverIliescu, CiprianRamos, Helena Maria Dos SantosAmin, Moeness G.Imamura, HiromiRampersad, SephraAmin, M. AshrafulInalpolat, MuratRamsey, BenjaminAmin, MohammadInfantino, AlessandroRana, Md. SohelAmineh, Reza KhalajIng, JamesRana, D.Amiruzzaman, MdIngalls, ToddRanstam, JonasAmo-Ochoa, PilarIngber, LesterRantz, Marilyn J.Amodio, Maria LuisaIniesta, JesúsRao, AshwinAmor, Boulbaba BenInoue, MRaparelli, TerenzianoAn, WeiIodice, AntonioRapp, MichaelAn, BeongkuIoppolo, TindaroRaquet, John F.Anagnostopoulos, ChristosIosifidis, AlexandrosRasmussen, JesperAnastasiu, Hrisdtos Iossifidou, EleniRattfält, LindaAndersson, BengtIqbal, UmarRau, JiannyeouAndò, BrunoIrato, PaolaRauber, Thomas W.Andrei, OctavianIsada, TomonoriRaucoules, DanielAndreoni, GiuseppeIsakov, DmitryRawassizadeh, RezaAndrieux, FabriceIsern, DavidRawat, Danda B.Anfossi, LauraIsern-González, JosepRawat, PriyankaAngelidis, Pantelis A.Ishida, HiroshiRazansky, DanielAnghel, AndreiIshihara, SusumuRazaque, AbdulAngrisano, AntonioIshii, Renato P.Razionale, ArmandoAnker, Jeffrey N.Ito, YosukeRazzaque, Mohammad AbdurAnnaheim, SimonIwai, DaisukeRebaudengo, MaurizioAnnamdas, Venu Gopal MadhavIwasaki, AkiraReddy, YenumulaAnnett, MichelleIwasaki, YoichiroRedmond, StevenAnslyn, EricIyota, TaketoshiRedondi, Alessandro E. C.Anthimopoulos, MariosIzdebski, FraukeReed, Matthew PAnthony, Carl J.Izu, NoriyaReed, WayneAntoni, JérômeJaballah, Wafa BenReed, NickAntonio, John K.Jackson, NathanReeves, Stanley J.Antonopoulos, AngelosJacobsen, KarstenRehse, Steven J.Antunes, PauloJaffrezic-Renault, NicoleReich, OliverAnvar, Amir MohammadJaganathan, ArunReichard, Karl M.Aoyagi, TakahiroJaime, Castillo-LeónReichardt, ChristianApedo, Komla LolonyoJaksic, VesnaReichel, Erwin KonradAphale, Sumeet S.Jakubik, Wiesław P.Reig, CàndidApostol, DanJakubikl, W.P.Reinhardt, WolfgangAquino, ArturoJames, Daniel A.Reinoso, OscarArafa, AhmedJan, Shau-ShiunReis, Manuel J.C.S.Arami, ArashJang, LipingReis, NunoArandjelović, OgnjenJang, Ho WonReis, Boaventura F.Arasaradnam, Ramesh PJang, YeongminReiss, AttilaArash, BehrouzJanik, Leslie J.Reisslein, MartinAraujo, AlvaroJannin, SamiRejas, Juan GregorioAraújo, Aurelio.l.Jantunen, ErkkiRen, LiyongArbiol, JordiJara, AntonioRen, DahaiARCHETTI, Francesco AntonioJara, Carlos A.Ren, MeiqiArdenne, Arnold vanJarén, CarmenRenard, Pierre-YvesArduini, FabianaJaume-i-Capó, AntoniRenaudin, ValerieArgus, DonaldJavier, Francisco OtamendiRenault, EricAricò, PietroJavier, García-PintadoRendón Mancha, Juan ManuelAriese, FreekJe, ChangsooRenevey, PhilippeAriga, KatsuhikoJedelsky, JanReshetilov, Anatoly N.Aristodemo, FrancescoJedermann, ReinerRettberg, AchimAriyur, KartikJedrzejewska-Szczerska, M.Retterer, Scott D.Arjunan, Sridhar PoosapadiJee, Gyu-InRevel, Gian MarcoArnaboldi, ValerioJemielniak, KrzysztofReverdy-Bruas, NadegeArnon, ShlomiJen, Cheng-LungRexford, JenniferArribas, JavierJeng, Chyuan-hwanReza, Ahmed WasifArriola, AJensen, B. D.Rezaniakolaei, AlirezaArtese, G.Jeon, Eui-ChanRhudy, MatthewArth, ClemensJeon, DoyoungRiazuelo, LuisArtiemjew, PiotrJeon, HyoungsukRibés, A.Arvaneh, MahnazJeon, MinyongRicciardi, SerenaAslam, Dr. NaumanJeong, Hae-Duck JoshuaRichelli, AnnaAslan, KadirJeong, Dae HongRichiedei, DarioAsorey-Cacheda, RafaelJerath, KshitijRiedel, TillAsteriadis, Stylianos (Stelios)Jessop, Julie L.p.Riedl, MatthiasAstráin Escola, José JavierJi, LuyanRiedo, AndreasAtalay, OzgurJi, LeiRienow, AndreasAtamturktur, SezJi, ZhanlinRiggs, Brian C.Atchison, JenniferJi, HaifengRinaldi, StefanoAtia, MohamedJia, YunfangRincón, FernandoAtkinson, Gary M.Jia, XiupingRingdahl, OlaAttila, FazekasJia, JiuhongRinge, EmilieAttoh-Okine, Nii O.Jiang, XiaoningRios, RubenAudet, SamuelJiang, BrendanRipka, PavelAuvinet, EdouardJiang, HaoRishpon, JudithAvdikos, GeorgeJiang, RuinianRitchie, GlenAvino, SaverioJiang, WeiRitterath, MartinAvolio, AlbertJiang, YifeiRivadeneyra, AlmudenaAvramov, Ivan D.Jiang, ZhiliangRivadeneyra-Torres, AlmudenaAw, K.C.Jiang, LanRiveiro, BelénAy, ChyungJiang, NingRiziotis, ChristosAy, SuatJiang, HongboRizzini, Dario LodiAyala, Julian M. EstudilloJiang, XiaozhenRobbins, KayAzam, Saeed EftekharJiang, JunhuaRoberts, CraigAzar, MassoodJiang, XiaohongRobertson, William M.Azarderakhsh, RezaJiang, HongkaiRobertson, Evan G.Azkune, GorkaJiao, LeiRocca, FabioAzpilicueta, LeyreJiao, ZongxiaRocha, L.A.Azzari, GeorgeJimenez, CeciliaRocha, LuisBabiarz, ArturJiménez, Víctor P. GilRocha Neto, Ajalmar R.Baca, ArnoldJimenez, FelipeRocha-Santos, Teresa A.Bade, DirkJiménez-Jorquera, CeciliaRoda, AldoBader, MarkusJin, HaoRodenas-Herraiz, DavidBae, Young-chulJin, JiongRodger, James A.Bae, HyungdaeJin, XinRodgers, Geoffrey W.Baeg, Seung-hoJin, HongxiaoRodic, AleksandarBaena, Rodriguez YJin, DongRodrigues, AlirioBaer, Chris-tophJin, SunggeunRodríguez, Manuel DíazBahillo, AlfonsoJin, TaeseokRodriguez-Gonzalvez, PabloBahrami, FarshidJin, MiaoRoerdink, MelvynBai, XiangzhiJin, YichaoRogatkin, DmitryBai, OuJin, ZhigangRogier, HendrikBai, JinheJin, Yu-fengRognini, G.Bai, JinboJing, LeiRoh, Kyoung-MinBailey, Ryan C.Jirka, SimonRoh, ChanghyunBainbridge, ScottJiu, BoRohac, JanBajger, MariuszJizhou, LaiRohani, MohsenBajo, JavierJo, HongkiRojas, Alexander MalaverBakalov, PetkoJo, Wonuk (Josh)Rolfe, PeterBaker, TharJohannsmann, DiethelmRolfe, Stephen AlexanderBakuzis, Andris F.Johanson, UrmasRoman, Christopher Balandin, SergeyJohansson, ChristerRoman, RodrigoBalasis, GeorgiosJohn, DineshRoman-Rangel, EdgarBalasubramanian, KannanJohn, VijayRomani, AldoBalbinot, AlexandreJohnson, ColinRomero, GemaBaldi, AntonioJohnson, Blake N.Romero Troncoso, René De J. Baleizão, CarlosJohnson, DavidRomić, MarijaBalleri, AlessioJohnson, ThienneRoncella, RiccardoBalzter, HeikoJohnson, Nigel PRoning, JuhaBan, TakayukiJones, Scott B.Roos, WouterBaños Navarro, RaúlJones, Reese E.Ros Lis, Jose VicenteBanville, SimonJones, John D.Rosa, JoaquínBaraban, LarysaJones, Alex K.Roscher, RibanaBaraldi, PieroJones, James PeytonRoselli, LucaBarazzetti, LuigiJong, Gwo-jiaRosenberger, A.T.Barbe, KurtJoo, Sang-WooRosolem, Joao BatistaBarberis, AntonioJorgensen, Palle E.T.Rossi, René M.Barbosa, Carlos HallJouanneau, SulivanRossi, Pierluigi SalvoBarchiesi, DominiqueJoubert, StephanV.Rossi, René MichelBardong, JochenJournaux, LudovicRossi, StefanoBarki, HichemJovanov, EmilRossignol, J.Barmenkov, YuriJu, HeongkyuRotenberg, NirBarolli, LeonardJuang, Jyh-ChingRoth, BernhardBarone, Pier MatteoJuang, Jehn-YihRothkrantz, LeonBarr, ChrisJun, YongseokRothwell, Edward J.Barralon, PierreJuncker, DavidRoufarshbaf, HosseinBarranco, FranciscoJung, HyunyoungRousseau, DavidBarreda-Ángeles, MiguelJung, Hyung-SupRowe, W. BrianBarrettino, FaridJung, ChangyongRowe, RichardBarrientos Cruz, AntonioJunior, Alair DiasRoy, Serge H.Barriga-Rivera, AlejandroJuvancic-Heltzel, JudithRoy, AlanBarroso, MargaridaJwo, Dah-JingRozlosnik, NoemiBartlett, RebeccaKaasalainen, SannaRuan, HaowenBartolucci, MarcoKacem, NajibRuano, AntónioBaruzzi, AuroraKadone, HidekiRUBA, MirceaBarzaghi, RiccardoKaewunruen, SakdiratRuckdeschel, PeterBascetta, LucaKaftandjian, ValérieRuckelshausen, ArnoBashkatov, Alexey NikolaevichKagawa, TakahiroRückert, UlrichBaskoutas, SotiriosKagawa, KeiichiroRuddell, DarrenBaston, ChiaraKahrizi, MojtabaRudnitskaya, AlisaBastos, AlexandreKaiser, JozefRuegamer, AlexanderBastos, LuisaKaivosoja, JereRuehrup, StefanBatou, AnasKalantar-zadeha, KouroshRuello, GiuseppeBatur, C.Kale, Prof. Girish M.Ruggeri, GiuseppeBauer, MartinKAM, Chi ShanRühmer, DennisBaum, MarcusKamienkowski, J. EstebanRuhtz, ThomasBaumann, H.Kamijo, ShunsukeRuichek, YassineBauwelinck, JohanKaminska, BozenaRuiz, Jordi-roger RibaBayer, KarlKampel, MartinRuiz Fernández, Luís ÁngelBayoumi, MagdyKamruzzaman, MohammedRuiz-Ascencio, JoséBaz, Didier ElKamsu-Foguem, BernardRuizenaar, MarcelBazaei, AliKan, TetsuoRuo, YuanBeales, Paul A.Kanatas, Athanassios G.Ruotsalainen, LauraBeaudoin, ChrisKang, HangbongRuppen, AndreasBeaulieu, LucKang, Dong-joongRusciano, GiuliaBeck, James L.Kang, Byong-hoRuzgas, TautgirdasBecker, Leandro BussKang, Soon JuRyu, Seok ChangBeckwith, RichardKang, InpilRyu, DonghyeonBednarik, RomanKangas, MaaritS. Watson, WilliamBegg, RezaulKankanhalli, Mohan S.Sa, InkyuBegum, ShahinaKannan, RajgopalSaavedra, HaroldBeheim, Glenn M.Kanno, YuichiroSabatini, AngeloBeigl, MichaelKaradaglic, DejanSabatini, RobertoBeisl, UlrichKaramat, TashfeenSablatnig, RobertBelagiannis, VasileiosKarczmarek, PawełSabol, DonaldBelcher, Dr. Warwick J.Karim, Karim S.Sacchi, ClaudioBelden, JesseKarimi, HassanSadeghi, SeyedBelkin, ShimshonKarlgaard, Christopher D.Sadhu, AyanBellavista, paoloKartsakli, ElliSadjadi, FiroozBellido, Francisco J.Karvonen, HeikkiSadowski, ŁukaszBelmonte, OscarKashani, Alireza G.Sae-Bae, NapaBen Amar, AchrafKashevnik, A. MikhailovichSaeed, KhalidBender, PaulKasneci, EnkelejdaSaeed, YahyanejadBender, FlorianKatakis, IoanisSaeedi, SajadBendig, J.Katasonov, ArtemSagle, LauraBenedetti, MatteoKateris, DimitriosSaha, Swetank KumarBenezeth, YannickKatikou, PanagiotaSahajpal, RitvikBenito-Lopez, FernandoKato, TeruyukiSahoo, Suban K.Benmeddour, FaroukKato, FumitakeSahoo, Prasan KumarBennett, Bradford C.Kato, NeiSainz, BeatrizBennetts, Victor Hernandez Katsura, SeiichiroSakaki, TaisukeBenois-Pineau, JennyKatunin, AndrzejSakkalis, VangelisBenrhouma, OussamaKatupitiya, JayanthaSalamí, EstherBera, AniketKatz, Brian F.g.Salazar-Torres, Jose J.Berberich, JasonKaur, SarabjotSaleem, KashifBerezin, Mikhail Y.Kawarazaki, NoriyukiSaleh, RaadBergasa, Luis M.Kaya, TolgaSalem, OsmanBergeot, NicolasKeating, AdrianSales, SalvadorBerger-Vachon, ChristianKedzierski, MichalSales, JorgeBergeret, EmmanuelKee, ChangdonSalowitz, NathanBergmann, NeilKefauver, Shawn C.Salz, PeterBergmann, OlafKeller, EricSalzenstein, PatriceBergonzo, PhilippeKelly, Ryan T.Samczyński, PiotrBeriáin, AndoniKelly, JonathanSamek, WojciechBerkovic, GarryKelly, KevinSamou, SergeBERMAK, AMINEKelner, JudithSampalli, SrinivasBernards, MattKemp, Andrew H.Sampietro, DanieleBerneschi, SimoneKer, Jun-ingSan-Segundo, RubénBerretti, StefanoKeränen, KimmoSanayei, MasoudBertel, SvenKerman, KaganSancenón, FélixBertocci, FrancescoKeynton, RobertSanches, João M.Bestak, RobertKhamis, KieranSánchez, María-teresaBétaille, DavidKhan, Adil MehmoodSánchez, Jorge AntonioBetke, MargritKhan, UsmanSanchez-Espeso, PabloBevly, DavidKhan, SaadSanchez-Rodriguez, DavidBeyerer, JürgenKhan, Jamil Y.Sanctis, Mauro DeBhattarai, KeshavKhan, ZahoorSanderson, AllenBhatti, PamelaKhandelwal, ManojSandhu, AdarshBhuiyan, Sharif M. A.Khennane, AmarSandrasegaran, KumbesanBhuiyan, Mohammad Zahidul H.Khondoker, RahmatullahSandu, CorinaBi, GuoanKiang, Jean-FuSangelaji, BahramBiagi, LudovicoKiełczyński, P.Sanjust, EnricoBian, KaiguiKieninger, JochenSankaranarayanan, Aswin CBibbo, DanieleKilgore, KevinSanna, AndreaBicocchi, NicolaKillgore, JasonSansone, CarloBie, RongfangKilroy, Joseph P.Sant’ Anna, AnitaBien, FranklinKim, WonjunSantana, PedroBifulco, Gennaro NicolaKim, SoheeSanthanagopalan, SunandBigler, EmmanuelKim, UksunSanti, FabrizioBill, RalfKim, DukhyeonSantos, Daniel Rodrigues dosBirch, David M.Kim, SunghoSantos, PauloBirch, BrianKim, Seung-WooSantos, Filipe Neves DosBischof, Hans-PeterKim, YongSantos, AbelBisio, IgorKim, Young-KeeSantos Peña, MatildeBissacco, GiulianoKim, Joo-YoungSantulli, CarloBiswas, PradiptaKim, Mi JeongSanyal, JibonanandaBitelli, GabrieleKim, Sang WooSanz, J.Björninen, ToniKim, SangchoonSanz, PedroBladimir, BaccaKim, Dong-jooSappa, Angel D.Blakely, Jonathan N.Kim, HwagyunSappok, AlexanderBlanc, SaraKim, MoonseongSarabia, Esther GonzálezBlanch, JuanKim, AyoungSardini, EmilioBlankenbach, JörgKim, JaehwanSarigiannidis, PanagiotisBlas, Javier GarciaKim, Seok-minSarkar, SoumikBlaszykowski, ChristopheKim, KihyunSasidhar P. S., KalyanBlayo, AnneKim, Seong JinSatorres, Silvia MartínezBloisi, DomenicoKim, DonghyunSattar, FarookBoano, CarloKim, YoungsungSattler, TorstenBoedecker, GerdKim, YoohwanSauerwald, TilmanBogdan, PaulKim, Chang-SeiSaunders, JeffBogdanowicz, RobertKim, Jihyungsause, MarkusBogusz, JanuszKim, ChrisSausen, Paulo SérgioBöhl, EberhardKim, JongdeokSavage, NickBöhm, JohannesKim, ShinaeSawyer, ShaylaBöhringer, Karl F.Kim, Young L.Saxena, NavratiBoluk, YamanKim, Nam-SikSayago, IsabelBonaci, TamaraKim, Sang-JaeSberveglieri, VeronicaBond, Tiziana C.Kim, Kwang TaekScaioni, MarcoBonenberg, LukaszKim, KiilScalenghe, RiccardoBonestroo, WilcoKim, Du YongScalise, LorenzoBonin-Fon, FranciscoKim, Jung-TaeScamarcio, GaetanoBonnabel, SilvereKim, KihwanScarano, GaetanoBonnet, VincentKim, ChangjaeSchaal, StefanBonter, DavidKim, Jai-HoonSchade, SvenBorecki, MichalKim, Hyun-sungSchaefer, Eike OliverBorges, Paulo V. K.Kim, SunwookSchäfer, ThomasBoriga, RaduKim, Nam-YoungSchatz, Bruce R.Borisov, SergeyKim, KeekyoungSchauer, ThomasBorri, SimoneKim, Jung-HoonScheffler, Tracy L.Bos, LieweKim, Byeong-WooScheider, SimonBosche, FredericKim, Choong-unScheller, MaikBoschetti, GiovanniKim, Young OkScheller, FriederBoss, EmmanuelKim, YoudanSchena, EmlianoBotella, GuillermoKim, YounghyunSchildbach, GeorgBotzheim, JánosKim, KeonwookSchilling, MalteBouali, MarouanKim, Yoon YoungSchilt, StephaneBouchard, KevinKim, Hyung WonSchirhagl, RomanaBoudon, FrédéricKim, Jong SuengSchirrmann, MichaelBoudraa, Abdel-OuahabKim, Joo-HyungSchlindwein, Fernando SoaresBouman, JohannesKim, Kyeong-HwaSchmid, SilvanBOURCIER, JOHANNKim, Dong-sungSchmid, NataliaBourdon, PierreKim, Seon JooSchmid, UlrichBouroche, MelanieKim, Gil-HoSchmidhalter, UrsBOUWMANS, ThierryKim, Do YoungSchmidt, UweBovee, Toine F.H.Kim (Prof.), E.Schmidt, AdamBoyali, AliKinet, DamienSchmitt, CorinnaBrabazon, DermotKing, MariaSchmitt, RobertBrace, ChrisKing, Chung-taSchmitt, MartinBračun, DragoKirkko-Jaakkola, MarttiSchmitz, AlexanderBraga, Roberto AlvesKirsanov, DmitrySchnass, KarinBratov, AndreyKirste, TSchneider, Jörg J.Braun, TorstenKita, NobuyukiSchön, SteffenBravo, JoseKiyokawa, KiyoshiSchrapp, MichaelBravo, MikelKlaoudatou, EleniSchreier, GünterBrayner, AngeloKlar, AssafSchubert, MarkusBrazaitytė, AušraKleinebecker, TillSchultz, StephenBrennan, ThomasKlenk, JochenSchulz, RuthBrennan, Robert W.Klessig, HenrikSchulze, HolgerBrewer, PeterKlinker, GudrunSchumacher, ThomasBrewin, RobertKloft, MariusSchumacher, JensBridge, EliKluth, PatrickSciammarella, FedericoBridle, HelenKnechtle, BeatScioscia, FlorianoBriggs, MartinKnight, ChristopherScorilas, AndreasBrilakis, IoannisKnopp, DietmarScotney, BryanBrillante, LucaKnowles, JohnScudiero, EliaBrito, PauloKo, Li-weiSebastian, Jose MaríaBrock, StefanKo, Nak YongSebastiano, FabioBrolly, MatthewKo, ByoungChulSebök, ÉvaBrooks, Richard R.Ko, HyounghoSecchi, PiercesareBrooksby, Paula A.Ko, Chia-NanSedano, FernandoBröring, ArneKo, HoonSedlmeir, FlorianBrosseau, ChristaKobayashi, MakikoSegawa, HiroyoBrouzes, EricKocur, DusanSegura-Quijano, Fredy Brown, BrianKodera, NoriyukiSeibold, UlrichBrownjohn, James M.W.Koenders, LudgerSeitz, RudolfBrownlee, SandyKoene, AnsgarSekercioglu, AhmetBruggemann, TroyKoenig, KeithSekiya, HirooBruggi, MatteoKoh, JaehanSelyanchyn, RomanBrunelli, Davide Kohut, PiotrSemenova, YuliyaBrunner, Andreas J.Koien, Geir M.Semprebon, CiroBruno, JohnKoinkar, PankajSendra Compte, Dr. SandraBruno, FabioKõiva, RistoSenesi , Giorgio S. Bruno, RaffaeleKokkinos, PanagiotisSengupta, DipankarBruno, BarbaraKolski, ChristopheSenthilkumar, K.Bruschi, PaoloKomenda, AntonínSenwo, ZacharyBrutti, AlessioKondis, Lisimachos PaulSeo, HwajeongBryson, M.Kong, Ling BingSeppa, Ville-PekkaBu, XianheKong, De-mingSequeira, JoãoBu, LeiKong, Seung-HyunSequeira Gonçalves, Paulo JorgeBubola, MarijanKong, WanzengSerôdio, CarlosBuchinger, SebastianKong, JinmingSeung-Bok, ChoiBuchmann, ErikKönig, PeterSeveri, StefanoBuchowski, Maciej S.Konopsky, ValerySeviour, ThomasBuckley, Erin M.Konstantinou, NikolaosSexidis, Lazaros A.Budgett, DavidKonstantopoulos, C.Shaalan, N.m.Budzik, GrzegorzKoo, Ki YoungShabayek, RahmanBugdol, MarcinKoop, Gerritshafiq, muhammadBui, NicolaKopačková, VeronikaShah, PuravBuller, MarkKoplitz, BrentShah, Shishir K.Bullon, PedroKopsaftopoulos, F.P.Shaker, GeorgeBuonocore, FrancescoKoren, KlausShan, ChongxinBurian, AdinaKorostynska, OlgaShandarov, V. M.Burleigh, ScottKORPEOGLU, IbrahimShang, RonghuaBurns, Ryan D.Korposh, SerhiyShangguan, LongfeiBurrascano, PietroKortelainen, JukkaShao, Hong-BoBurrows, SusanKorvink, Jan G.Shao, LiyangBush, Brian WKoshiba, MamikoShapter, Joe G.Bustamante, RogelioKosmas, PanagiotisSharifi, Farrokh J.Butkiewicz, ThomasKosmopoulos, DimitriosSharma, Suresh C.Buttafuoco, GabrieleKostopoulos, VSHARMA, Shree KrishnaButtazzo, Giorgio C.Kotsiantis, SotirisSharma, Piyush SindhuBüttgenbach, StephanusKotze, BenShaw, Geoffrey M.Caballero-Gil, CándidoKoulamas, ChristosShaw, Kirsty J.Cabello, EnriqueKoulouriotis, D. E.She, QingshanCabeza, RafaelKouroussis, GeorgesShen, LinlinCabrera, Juan AntonioKoutsoudis, AnestisShen, YuzhongCaduff, RafaelKoutsoukos, XenofonShen, YanfengCaetano, E.Kouzoudis, DimitrisShen, YaochunCahalane, ConorKovatsch, MatthiasShen, JuCai, ZhongyuKownacki, CezaryShen, WeiCai, YinghaoKraft, MartinShen, ShaojieCai, JinhaiKrajewski, JarekShen, Tsu-WangCai, BaopingKrasnov, Oleg A.Shen, Sheng-ChihCalbimonte, Jean-PaulKrause, ThomasSheng, MichaelCaldara, RobertoKrawczyk, BartoszSheng, Guo-PingCaldeira, João M. L. P.Kreindler, LiviuSheng, DuoCaleffi, MarcelloKrejcar, OndrejSheng, ZhengguoCallahan, Laura SmithKrishnan, SadagopanSheng, BinCallaway, Andrew J.Krishnan, SridharShepherd, PeterCallegari, SergioKristensson, Per OlaSherkat, NasserCalleja, MontserratKronberger, RainerShi, ZhenweiCallejas, ZoraidaKruell, WolfgangSHI, ZUOQIANGCalligaris, SoniaKrüger, BjörnShi, PengCalveras, AnnaKruger, AntonShi, JunCaminero, M.A.Krusienski, DeanShi, JingCampa, AlessandroKsiezopolski, BogdanShi, YaochengCampagna, MicheleKu, ZahyunShi, YangCampas, MonicaKuang, Cui-fangShi, JunboCampbell, CarleneKubičár, ĽudovítShiang, Tzyy-YuangCampbell, ColinKuc, Tae-YongShiau, Jaw-KuenCampbell, JanetKudela, PawełShibata, YuichiroCamplani, MassimoKugiumtzis, DimitrisShieh, Wann-YunCampo, EricKuhlmann, Heiner Shih, Wen-PinCampopiano, StefaniaKühnicke, ElfgardShih, Po-JenCampos, RicardKuipers, BenjaminShih, Tian-YuanCampos Rubio, Juan CarlosKulathumani, VinodShih, Ching-longCampuzano Ruiz, SusanaKumar, PardeepShim, Yoon-BoCamtepe, SeyitKumar, PankajShimabukuro, YosioCañadas Quesada, Francisco JesusKumar, SumanShimizu, ToshimiCañete, EduardoKumatani, KenichiShimizu, YukiCanuto, EnricoKun, Andrew L.Shimshoni, IlanCao, KaiKuno, YoshinoriShin, Chow-ShingCao, CuongKunz, SebastianShin, Soo-YoungCao, HaishiKuo, Chung-HsienShin, Jae HoCAO, ZhuoKuo, Chil-ChyuanShin, J.T.Cao, MaosenKuo, Chung-yenShin, HainsworthCapella Hernández, Juan VicenteKuo, Chih-YuShintaku, HirofumiCapello, ElisaKurabayashi, DaisukeShiraishi, YasuhiroCapilla, RafaelKurazume, RyoShirasaki, YoshitakaCapineri, LorenzoKurbatov, A.M.Shoji, YoshinoriCapovilla, Carlos E.Kuremoto, TakashiShokouhi, ParisaCAPPA, PaoloKuroda, RihitoShorter, Joanne H.Cappiello, CinziaKurt, MehmetShortis, Mark RichardCaprari, GillesKusyk, JanuszShoshany, MaximCapuano, RosamariaKwak, Kyung SupShu, XuewenCarabelli, V.Kwak, Moon K.Shull, Pete B.Cardei, IonutKwok, Ngai MingShum, Hubert P. H.Cardoso, SusanaKwon, Oh-KyongShyrokau, BarysCardoso, JaimeKwon, Ji-WookSi, Xiao-ShengCardullo, Richard A.Kwon, Jae KyunSibielak, MarekCarmo, JoãoKwong, Chan PakSicard, GillesCarpenter, Michael A.Kyba, ChristopherSicari, SabrinaCarrascosa, Laura GarciaKytomaa, Harri K.Siddiqui, Mohummad MinhajCarrera, ErasmoLa, Hung ManhSidibé, DésiréCarrillo Calleja, Juan ManuelLa Cour-Harbo, AndersSidiropoulos, PanagiotisCaruso, AnthonyLabati, Ruggero DonidaSiegel, MelCasale, MonicaLaborde, RomainSigl, GeorgCasas, Joan R.Laboski, CarrieSijs, JorisCasciati, FabioLachapelle, GérardSikes, HadleyCasciati, SaraLackovic, IgorSikorski, WojciechCasilari Pérez, EduardoLade, HarshadSillanpaa, MikaCasini, MarcoLadikos, AlexanderSilva, LucianoCassidy, NigelLagkas, ThomasSilva, AlejandraCasson, Alexander J.Lago, Claudimir Lucio DoSilva, HCastilla, MariaLAGRANGE, XavierSilva, Rone Ilídio daCastrillón-Santana, ModestoLagüela, SusanaSilva, IvanovitchCastro, Rebecca MirandaLai, Chao-SungSilvester, Debbie S.Castro-Hurtado, I.Lai, JianjunSim, Sang JunCatalina, TiberiuLai, Shang-HongSim, S.H.Catarino, AnaLai, Andy Shui-YuSimani, SilvioCaumon, Marie - CamilleLai, Ying-ChihSimic, MilanCavallini, MassimilianoLaidlaw, Stephen M.Simó, JoséCavallini, AndreaLajimi, S. Amir MousaviSimon, WilliamsCawley, PeterLakner, HubertSimonescu, Claudia MariaCazzaniga, Noemi E.Lallart, MickaëlSimpson, Garth J.Cazzulani, GabrieleLam, Wilbur A.Sims, NeilCecil, J.Lam, Kwok-hoSin, MandyCecotti, HubertLambert, Andrew J.Singh, Kunwar K.Cela, Sara AbaldeLamberti, FabrizioSingh, DhananjayCellini, NicolaLamonaca, F.Singh, KanwarpalCennamo, NunzioLampoltshammer, Thomas J.Singh, RameshCeriotti, MatteoLanceros-Mendez, S.Singhal, MukeshČernÝ, MartinLandaluce, HugoSio, Luciano DeCerra, DanieleLanders, AndrewSipal, VitCerretani, LorenzoLang, Hans PeterSirakov, NikolayCervantes, JairLang, JochenSisinni, EmillianoCesano, FedericoLang, JeffreySismaet, HunterCesarini, DanielLarijani, HadiSiu, Kin Wai MichaelCevenini, LucaLarrarte, FrederiqueSkomorowski, MarekCha, YoungsuLascala, Cesar AngeloSladek, JerzyChaaraoui, Alexandros AndréLaskowski, RafalSlinker, Jason D.Chabot, DominiqueLasky, TySlobodian, PetrChae, YoungcheolLatré, StevenSmarsly, KayChae, HeeyeopLattanzi, GianlucaSmeaton, AlanChai, (raullen) QiLattuada, MarcoŠmíd, Radislavchai, yatingLau, LawrenceSmietana, MateuszChakraborty, SubhadeepLau, MichaelSmirnov, AlexanderChalhoub, GerardLaurenzis, MartinSmith, DavidChalmers, SharonLaut, Jeffrey W.Smith, StuartChamanzar, MaysamrezaLavine, BarrySmith, KandlerChan, Chia-TaiLavrentovich, Oleg D.Smith, EmilyChan, Warren C.W.Law, Wing-CheungSmith, JasonChan, MatthewLaw, Yee WeiSmithard, DavidChan, Garylay ekuakille, aimeSmolina, IrinaChandler, Jim H.Laza, RoslíaSmolinska, AgnieszkaChandrasekar, V.Lazar, JosefSmolka, Scott A.Chang, Chih-yuanLazarakis, FotisSmulko, JanuszChang, Chih-WeiLazarescu, Mihai TSnape, JamieChang, Shao-HsiaLazaro, AntonioSnee, PrestonChang, Rwei-ChingLázaro, JesúsSo, Hing-cheungChang, JiyulLazzeri, RobertaSoares, Vasco N.G.J.Chang, Cheng-ChunLe, QiangSoares, Luis DuclaChang, Pei-ZenLe Bihan, NicolasSoh, Cheong BoonChang, Jhih-ChungLe Meins, Jean-FrançoisSoh, Gim SongChang, Tong-BouLe Pen, LouisSohel, FerdousChang, Yao-JenLeccese, FabioSohgawa, MasayukiChang, Chung-LiangLee, Sang-GonSohn, HonglaeChang, Hsueh-HsienLee, Moon HoSohn, Young-sooChang, Kang-MingLee, SungyoungSolano, Juan P.Chang, Ray-iLee, In-KwonSolari, FabioChang, Chih-WenLee, ByounghoSoleimanpour, Amir MasoudChang, Tae GyuLee, DavidSoliman, MohamedChang, HonglongLee, TaekkyeunSolimene, RaffaeleChang, Kuang-YuLee, Sang HunSoloviev, MikhailChang, IPLee, Chao-hsienSolovieva, YuliaChao, YangLee, Woo-KyungSoltanieh, Mohsen KalantariChao, Benjamin F.Lee, Cheng-LingSong, LijunChapeleau, X.Lee, Jung-WonSong, KaichenChapman, EliLee, JungchulSong, MinkyuCharki, AbdérafiLee, Yong WookSong, Yeong-taeCharleston-Villalobos, SoniaLee, Byeong HaSong, Taek LyulCharlton, Peter ChristopherLee, Wee SitSong, AiguoChatterjee, SnehamoyLee, JangmyungSong, YangChatzi, EleniLee, Ki YongSong, ZhanChatzichristidi, MargaritaLee, Ching-TingSong, KaiChau, YuenLee, JaySong, HoubingChau, Kwok-WingLee, GeehyukSong, HongChau, Lai-KwanLee, Seok JaeSong, WenjiaoChe, WeitaoLee, Chi-YuanSong, JaeSeungChehimi, MohamedLee, Hsiang-ChiehSongchitruksa, PraprutChellappa, RamaLee, JiyeSonnemann, T.F (Till)Chembo, Yanne K.Lee, Peter Peng FooSoon, Yeo TatChen, GangLee, Shie-jueSophocleous, MariosChen, XiyuanLee, SanghunSorge, ChristophChen, HongLee, KeekeunSoria, Silvia Chen, QiangLee, Yang-hanSoria Morillo, Luis MiguelChen, Yuan-TsungLee, TaeyoonSoriano, Julio GómezChen, NengchengLee, DongjinSorli, BriceChen, Der-chinLee, AhyoungSorniotti, AldoChen, Ping-HeiLee, Chun-YaoSośnica, KrzysztofChen, CailianLee, Dah JyeSoto, RogelioChen, Ching-MeiLee, SangukSoto, RicardoChen, LijiLee, Hung-KyuSoto Campos, IgnacioChen, JieLee, KijoonSouza, Paulo deChen, JianLee, D.-S.Spaccini, DanieleCHEN, Ben M.Lee, DongkyuSpachos, PetrosChen, WuLee, Chun-TaSpadea, Maria FrancescaChen, YuweiLee, DuckkiSpagnoli, AndreaChen, WeiminLee, Seung-WooSpagnolo, VincenzoChen, JunhongLee, MinsikŠpakov, OlegChen, Lin-ChiLee, Hoon-JaeSpanakis, Emmanouil GChen, HongyangLee, DonghwaSpataru, SergiuChen, XianfengLee, MinhoSpencer, Billie FChen, ChengciLee, Hsiao-YiSpicer, JamesChen, GuangLee, HungkyuSpina, MassimoChen, Lung-ChienLee, Hyunjoo J.Spinazzè, AndreaChen, Chao-HoLee, Sang-GugSpinsante, SusannaChen, Hui-fangLee, KyujinSpivak, ArthurChen, ShaxunLee, ChangnoSpizzichino, ValeriaChen, ShanbenLee, Tian-fuSporea, DanChen, WeiLee, Jong-HyoukSrinivas, UmamaheshChen, Shin-JuhLee, Jeong HoonSrinivasan, RengaswamyChen, YuanzhuLee, HyokiStacey, M.Chen, ShengLee, TaikjinStagni, RitaChen, Shih-YuLee, Ju-yiStanacevic, MilutinChen, TingtingLee, HeesungStanca, Sarmiza ElenaChen, Yen-LinLee, Ji-HoonStančin, SaraChen, YangkangLee, ChaBumStanica, RazvanChen, Lien-WuLee, JoonseokStansfield, BenChen, ErxueLee, Cheng-ChiStasiak, BartłomiejChen, AichengLee, Nae-EungStassi, StefanoChen, Yen-DaLee (Prof), S.Stateczny, Andrzej EugeniuszChen, XingLefeuvre, ElieStavrakas, IliasChen, QuanshengLei, YaguoStechele, WalterChen, WenLei, Ting WuSteenhaut, Ir. KrisChen, HaimingLei, YuStefani, StefaniChen, Chung-HaoLei, XushengStefanof, KonstantinChen, Bor-SenLei, JianpingStegagno, PaoloChen, Denis GuangyinLeicht, LennartSteinbach, EckehardChen, Shu-HuiLeinen, StefanSteiner, MatthewChen, Teng-KaiLeis, JohnStephenson, Sally-AnneChen, Kuan-NengLeitner, GerhardStergiou, DimitrisChen, RogerLeitzmann, MichaelSternberg, HaraldChen, XuedongLemaire, EdwardSteyn, WillemChen, JiajunLenzen, ChristophStiharu, IonChen, Jen-JeeLenzi, TommasoStirling, LeiaChen, YouganLeone, AlessandroStiros, Stathis C.Chen, Yen-LunLeppakoski, HelenaStobiecka, MagdalenaChen, KongzheLerchner, JohannesStone, Kenneth CChenele, DavidLerner, AmitStonehouse, NicolaCheng, XuanhongLeroux, DenisStork, WilhelmCheng, Chi-TsunLesiak, PiotrStowell, DanCheng, TengLeucci, GiovanniStoy, PaulCheng, ShunfengLeung, Christopher K. YStoyanov, DanailCheng, Wood-hiLeung, HowanStoykovich, Mark P.Cheng, Wei ChenLeva, SoniaStrano, Michael S.Cheng, Chia-HsinLevitskaia, Tatiana G.Strehlitz, BeateCheng, HongLevy, JosephStrelcov, EvgheniCheng, Juei-tangLevy, CesarStride, John ArronCheng, Chen-YangLi, Kwok HungStriley, CynthiaCheng, KaiLi, QingquanStrumiłło, PawełCheng, JingyuanLi, HengjianStrupinski, WlodekChestek, CindyLi, YangmingStuckenschmidt, HeinerChew, Chee-MengLi, NaSu, WencongChhetri, Mohan BaruwalLI, Jian-XunSu, JiaChiabrando, F.Li, Pei-WuSu, Ming-ShouChiang, Kai-WeiLi, Chang-MingSu, YanChiang, Cheng-TaLi, DiSu, XiaojieChiani, MarcoLi, Jun-BaoSu, Kuo-LanChiao, Jung-ChihLi, HaoSu, FeiChien, Ying-RenLi, ChangleSu, Mu-chunChih, MingchangLi, ShuguangSu, HongboChikashi, NakamuraLi, JingsongSu, XiangChikkadi, KiranLi, ShihuaSu, FulinChin, Craig A.Li, ZongzhiSu, Tung-ChingChin, Cheng SiongLi, Wen JungSu, RuoyuChing, Congo Tak ShingLi, WenzhongSu, Guo-Dung JohnChiolerio, AlessandroLi, FuqinSuarez, AlvaroChitnis, G.D.Li, jianxinSubramanian , Ananth Chiu, Tai-chiaLi, WeiSubramanian, NaryCho, Sung HoLi, LingyunSuchenwirth, LeonhardCho, KyungeunLi, QingSudduth, Kenneth A.Cho, Young ImLi, JunSudhölter, Ernst J.R.Cho, Jae YoongLi, NanSuess, DieterCho, JunghwanLi, QunSugano, ShigekiCho, GihwanLi, XiaoliSugano, MasashiCho, SoojinLi, JingSuh, Young SooCho, Ho-ShinLi, FeiSuh, Soo HwanCho, SungboLi, MingyuSuh, Doug YoungChoi, JunhoLi, ShuaiSuhling, KlausChoi, H.D.Li, DavidSui, XiaohongChoi, JongeunLi, YangSujit, P.Choi, Ji-WoongLi, ZhijunSullivan, BenjaminChoi, Jae-WonLi, HongdongSullivan, Rani W.Choi, Byong-DeokLi, WenjiaSullivan, Pierre E.Chon, Ki H.Li, YuheSummy, Kenneth R.Chong, AlvinLi, JianSumner, JoyChong, RaymondLi, ShancangSun, XiudongChoo, JaebumLi, ZhiSun, ShuliChoppala, PraveenLi, MeixianSun, QingquanChou, Kan-senLi, KaiSun, WeiChouvardas, Vasilios G.Li, QingguoSun, Nai-HsiangChouvion, BenjaminLi, HongkunSun, WenChow, JackyLi, QingyongSun, YunchuanChowdhury, MorshedLi, XiaopengSun, YuruiChowhdury, SazzadurLi, TianchengSun, Nian X.Chrcanovic, BrunoLi, Yen-FangSun, KewenChristos, AntonopoulosLi, HuanSun, WeiqingChu, Shu-ChuanLian, Feng-LiSun, HaixinChu, Hung-ChiLiang, LitaoSun, ChangmingChu, Cheng-shaneLiang, Jun-RuiSun, HongjianChu, XinzhaoLiang, YuSun, JieChuang , Kuo-ChihLiang, Chiu-kuoSun, ShiliangChuang, Cheng-LongLiang, XuefengSun, YeChuang, Po-JenLiang, XishuangSun, XiaoweiChuang, Wan-ChunLiang, GuolongSun, HongzhiChui, Hsiang-ChenLiang, HuaweiSun, Hong-TaoChun, Myung-SukLiang, YanSung, Guo-mingChun, SejongLiao, WenzhiSur, RitobrataCHUNG, D.D.L.Liao, LeiSuriadi, SuriadiChung, Ching-CheLiao, LinxiaSusi, MelaniaChung, Yu ChiLiao, GuanglanSuski, WilliamChung, SeungjunLiao, HongenSutor, AlexanderChung, In-HangLiao, Bin (Benny)Suykens, Johan A. K.Chung, Sun-okLiao, ShaolinSuzuki, Hiroaki Chung, HoeilLiatsis, PanosSuzuki, TaroChung, Tein-YawLiaw, Shien-kueiSuzuki, KenichiroChung, YeonHoLieberzeit, PeterSuzuki, YujiChung, Jae-HyunLiebig, ThomasSveda, MiroslavChuquet, JulienLight, GlennSvensson, ErikChusuei, CharlesLigorio, GabrieleSwapna Kumar, S.Ciaccheri, LeonardoLim, SeokbinSweetman, MartinCianca, ErnestinaLim, Jun-SeokSwillam, Mohamed A.Ciminelli, CaterinaLim, SungjoonSyrrokostas, G.Cintron, Fernando J.Lim, Dong-WonSysoev, Victor V.Cionca, VictorLim, Gi HyunSzabo, LorandCipresso, PietroLim, Yah Leng Szewczyk, R.Cirani, SimoneLin, Wei-YangSzmacinski, HenrykCiuonzo, DomenicoLin, Kuo ChiSzöllősi, DánielClarke, KeithLin, ChiSzumanowski, AntoniClarkson, SeanLin, SebastianSzunerits, SabineClaudel, Christian G.Lin, Pei-chunSzypłowska, AgnieszkaClausen, IngelinLin, Jiu-jengTabrizi, Ali AkbarClausen, PhilipLin, Hong-boTaffoni, FabrizioCleland, IanLin, YuqingTaheri, SaiedClemente, CarmineLin, Yuan-PinTaimre, ThomasClinton, SmithLin, TaoTajbakhsh, Shahriar EtemadiCocconcelli, MarcoLin, Bor-ShyhTakahashi, YasutakeCodetta-Raiteri, D.Lin, Chii-WannTakenaka, NorimichiCoelho, LuisLin, Bao-HongTakizawa, HotakaCole, MathewLin, Tian RanTalei, AminCollin, JussiLin, Chin-fengTalens, PauCollini, ElisabettaLin, BinTamás, LeventeCollotta, MarioLin, Lih Y.Tamborrino, AntoniaColosimo, B.M.Lin, Chih-mingTamura, ToshiyoColosimo, GabrieleLin, WeiTan, LianshengComolli, LorenzoLin, TsungnanTan, Yen KhengCondino, SaraLin, Chih-HongTan, Chee WeeCongedo, Paolo MariaLin, Yaw-jenTan, U-XuanConger, ScottLin, Chih-Ming (Gimmy)Tan, Say HwaConoci, SabrinaLin, Tzung-HanTanabe, TakasumiContreras, PedroLin, LinTanaka, ToshihisaCook, DuncanLin, BoTanaka, HiroshiCorato, Francesco DiLin, MichaelTancredi, UrbanoCorchado, Juan M.Lin, Hung-yinTang, FeiCorcoran, PeterLin, Ting-LanTang, HaoCordero Fuertes, Juan AntonioLin, Hsueh-chunTang, Fuk-hayCórdoba, RosaLin, JingTang, WeiCorigliano, AlbertoLin, Cheng-An JTang, LiqiongCorreia, FernandoLin, ChingShunTang, C. J.Correia, Jose HiginoLin, FengTang, XinhuaCortes, PedroLin, Che-HsinTang, JieCosta, NunoLin, Chia-ChenTang, K.t.Cotos, José M.Lin, GungunTang, YijunCottyn, JohannesLin, Huei-YungTang, JianCovington, JamesLin, Ming-TzerTang, Shi-YangCozzolino, DanielLin, Yih-HwangTao, LeiCozzolino, AnthonyLinden, MariaTao, LiliCracowski, J.l.Lindquist, Nathan C.Tao, JunliangCragg, Peter J.Ling, Yong-ChienTao, Michael W.Cramer, MichaelLingua, AndreaTao, DapengCrescentini, MarcoLinnhoff-Popien, ClaudiaTao, HaihongCretu, EdmondLipsett, MikeTapete, DeodatoCristina, Ana MajarenaLisotti, AndreaTapia, EmmanuelCrivelli, DavideLissenden, CliffordTarantino, EufemiaCroft, Paul J.Little, JimTardif, Pierre MartinCuadras, AngelLitton, Charles DTarel, Jean-PhilippeCubo, JavierLiu, ZipingTarricone, LucianoCuesta, FedericoLiu, yingyingTaseska, MajaCuesta González, EduardoLiu, YonggangTasis, DimitriosCugnoni, JoëlLiu, YangTavaré, Simoncui, songLiu, JindongTavares, AnaCui, YiLiu, DalongTavío, María del MarCulbertson, Christopher T.Liu, XinyuTedesco, PatriciaCULSHAW, BrianLiu, ChuanjunTee, BenjaminCunha, MárioLiu, HaiTegolo, DCunningham, ShawnLiu, Bing-hongTEH, Ying-KhaiCurado, MariliaLiu, QiongTeixeira, LuisCuriac, DanielLiu, JuTeixeira, FernandoCusano, AndreaLiu, LiangguiTelem, GiliCutkosky, Mark R.Liu, ZenghuaTeles, F. Sérgio Rodrigues Ribeiro D Lemaire, EdwardLiu, YongbinTelfer, BrianD'Amico, Arnaldoliu, HuiTelkki, Ville-veikkoD'Oleire-Oltmanns, SebastianLiu, XinxinTemple-Boyer, P.D'Orazio, TizianaLiu, FengTenbohlen, StefanD'Urso, GuidoLiu, XiaohanTenedorio, Jose AntonioDa Silvab, Joaquim C.g. EstevesLiu, HonghaiTeng, JunDabove, PaoloLiu, TaoTeng, DayuDagdeviren, OmurLIU, MingTeng, Shyh WeiDagdeviren, CananLiu, YanjunTennina, StefanoDahlin, AndreasLiu, JunyanTeo, Tee-AnnDai, YuchaoLiu, BifengTepe, Kemal Dai, JishengLiu, XuxunTeresa Sánchez, MaríaDai, FeiLiu, YunTestoni, NicolaDaigle, Courtneyliu, wcTeunissen, PeterDakins, MaxineLiu, XiaofengTeunissen, Peter. J. G.Dal Moro, GiancarloLiu, XinTeza, GiordanoDalla Betta, Gian-FrancoLiu, Jing-QuanThakor, NitishDamas, MiguelLiu, BinThalheimer, MartinDan, YapingLiu, WilliamTheodorakopoulos, IliasDanaher, TimLiu, HaoTheodoridis, EvangelosDanescu, Radu GabrielLiu, QingshengThibault, ChristopheDang, Duc Ngoc MinhLiu, JunThielemann, ChristianeDaniela, CarrionLiu, YingshuaiThirunarayan, KrishnaprasadDaniele, PasseriLiu, WeiThomson, Steven J.Daniele, MichaelLiu, BoThornell, GregerDaniels, StephenLiu, LichuanThostenson, ErikDanson, MarkLiu, WeitingThouin, LaurentDantcheva, AntitzaLiu, XintaoTian, JingDarehshoorzadeh, AmirLiu, GuigenTian, GuiyunDarrah, MarjorieLiu, ZhengTian, ZengshanDarvin, MaximLiu, JiangguoTian, YanqingDaskalopoulos, IoannisLiu, NanTian, GangDauwels, JustinLiu, AiqunTian, WenxiDavid, SpiererLiu, Chung-ChiunTian, ZhenhuaDavid Mascarenas, DavidLiu, HongyunTidoni, EmmanueleDavies, ClaireLiu, Yen-ChenTieve, HelioDavis, John PLiu, Yung-tienTigbe, WilliamDawidowicz, AgnieszkaLiu, JingweiTiitta, MarkkuDawidowicz, K.Liu, YongTimpe, Shannon JDe, SoumyaLlaves, AlejandroTina, Giuseppe Marcode Blas, MaiteLloréns, RobertoTjin, Swee ChuanDe Coninck, JoëlLloret-Mauri, JaimeTobe, YoshitoDe Cos, ElenaLloyd Spetz, AnitaTobing, Landobasa Y. M.De Dinechin, FlorentLo, Men-TzungTodoroff, PierreDe Fazio, MarcoLo, Chien-fongTodorovska, Maria I.De Kerpel, KathleenLöchtefeld, MarkusTognetti, Alessandrode la Escalera, ArturoLogsdon, Sally D.Tokuda, TakashiDe la Torre, ÁngelLoh, Tian HongTollefsen, DagDe Leonardis, FrancescoLoh, Chin-HsiungTollmar, Konradde Lima, Jr., Mauricio M.Lohan, Elena SimonaTollner, Ernest W.De Marcellis, AndreaLoia, VincenzoTomarchio, OrazioDe Rossi, Stefano Marco MariaLoke, Seng W.Tomas , MénardDe Spiegeleer, BartLombardi, John R.Tomassetti, MauroDe Vito, SaverioLong, D.A.Tomažič, SimonDebeda-Hickel, HélèneLongo, DomenicoTomkiewicz, DariuszDeBlois, FrancoisLongstaff, Andrew PeterTommaselli, Antonio M. G.Decho, AlanLopes, JoãoTondini, NicolaDecotignie, Jean-DominiqueLopes, LuísTong, YanDedele, AudriusLopez, Jose J.Tong, RuofengDee, Hannah M.Lopez, JuanTong, LiangDei, MicheleLópez, José MaríaTong, HanghangDeka, LipikaLópez de Ipiña, DiegoTonokura, KenichiDel Bue, AlessioLopez Fernandez, JoseTöreyin, HakanDel Din, SilviaLópez Villanueva, J. AntonioTörngren, MartinDelalieux, StephanieLopez-Bejar, ManelTörnqvist, DavidDelehanty, JamesLopez-de-Ipiña, KarmeleTorregrossa, DimitriDelele, MulugetaLopez-Higuera, Jose MiguelTORRENS, FranciscoDell’Olio, FrancescoLópez-Lorente, Á. I.Torres, DavidDella Corte, Francesco G.Lopez-Molina, C.Torres, MagdaDelmas, Jean PierreLopomo, NicolaTorres-Sospedra, JoaquinDelmastro, FrancaLoprinzi, PaulToschi, IsabellaDelnavaz, AidinLorenz, PascalTosi, DanieleDemeyer, PeterLorenzo, Rubén MTOTH, GezaDemirci, UtkanLori, Nicolás F.Toth, CharlesDemirkol, IlkerLos, SietseTothill, Ibtisam E.Demosthenous, AndreasLou, XiaTotis, G.Deng, ZhihongLou, WeiTouhafi, AbdellahDeng, Z. DanielLoutas, TheodorosTragos, Elias Z.Deng, ShuguangLoutridis , Spyridon Trahanias, PanosDeng, ZhengMiaoLovisolo, LisandroTran, Gia KhanhDéniz, O.Lu, PingTraversaro, SilvioDenman, SimonLu, GeyuTravieso González, Carlos ManuelDennis, AllisonLu, ChaoTravieso-González, Carlos M.Deravi, Leila F.Lu, Chia-JungTrebaol, StephaneDeschaud, Jean-EmmanuelLu, XiaodongTreiber, MartinDesertot, MikaelLu, LiyouTrifiletti, ADeshi, LiLu, XuejunTrigona, Eng. CarloDeshpande, AjayLu, WenlianTrimpe, SebastianDeslouis, ClaudeLucani, Daniel E.Trinder, JohnDesmulliez, MarcLuches, ArmandoTrizna, DennisDeSouza, GuilhermeLudwig, Dr. FrankTrogl, JosefDesreux, Jean F.Lufaso, MichaelTroisi, SalvatoreDetweiler, CarrickLughofer, EdwinTrommer, Gert F.Devitt, DaleLuhmann, ThomasTrusov, A. A.Devkota, JagannathLuís Berz, EvertonTsai, Meng-TsanDi Gennaro, Salvatore FilippoLuis Bustamante, AlvaroTsai, Wen-ChiDi Maio, RosaLuján, Jose Luis PozaTsakalof, ADiaconu, Adrian-ViorelLund, AnjaTsang, Kim FungDiamant, RoeeLuo, JunTse, Peter W.Diamond, DermotLuo, EricTseng, Tina T.-C.Dianat, PouyaLuo, YanhuaTsianos, NikosDias, KelvinLuo, Ching-HsingTsiftsis, Theodoros A.Dias, Luís G.Luo, XichunTsiknakis, ManolisDias, JorgeLuo, WeiTsiourlis, GeorgeDíaz-Vilariño, L.Luo, ChaominTsipouras, MarkosDickens, TarikLuong, John H. T.Tsow, FrancisDickert, Franz L.Luštrek, MitjaTsuboi, TaijuDietzel, StefanLützenberger, MarcoTsukada, KeijiDijksman, J. FritsLuukka, PasiTsuzuki, MarcosDilley, RodneyLuyckx, GeertTsytsarev, VassiliyDilo, ArtaLv, KeTu, RuiDing, Binlv, shaolinTuan, Chiu-chingDing, Jow-LianŁuczak, SergiuszTuan, GuoDing, DaleM. Callico, GustavoTuck, Kellie L.Ding, Yong-shengM. Close, DanTung Chong, David WongDing, ZhifengMa, XiangTuononen, Ari JuhaniDing, YanjunMa, AnzhouTurcza, PawelDing, YongMa, Guo-mingTurner, DarrenDios, J.r. Martinez-deMa, YangjinTutwiler, RichardDiraco, GiovanniMa, Der-MingTwomey, KarenDitmer, Mark A.Ma, YideTzallas, Alexandros Dixon, NeilMa, XingTzanetakis, GeorgeDjahel, SoufieneMa, PingTzeng, Yu-ChangDjamaa, BadisMa, LinUbertini, FilippoDobosz, MarekMa, JianguoUbertini, FrancescoDobre, CiprianMa, HuiUchida, TakahiroDodd, Ian C.MacDonald, BruceUeno, YukoDodd, Linzi E.MacGregor, M.H.Uhler, Kristin M.Dogaru, RaduMach, PavelUhring, WilfriedDöhler, MichaelMachida, MasashiUllah, SanaDolan, John M.Macias, ElsaUlrich, MarkusDolev, ShlomiMacias-Bobadilla, GonzaloUmek, AntonDoll, JoeyMacnae, James C.Uno, ShigeyasuDoménech-Carbó, AntonioMaddahi, YaserUrbanelli, LorenaDomenico, GrimaldiMaeda, IsamuUrbann, OliverDominguez, Aníbal RenonesMaehle, ErikUrbansky, RalphDomínguez, Juan Jesús GarcíaMagda, Jules J.Urdampilleta, MatiasDominguez Brito, Antonio CarlosMagee, JohnUreña, JesúsDominici, DonatellaMaglaras, LeandrosUsamentiaga, RubénDommermuth, FranzMagliulo, MariaUslander, ThomasDonati, Prof. SilvanoMagney, Troy SUslar, MathiasDonelli, MassimoMagno, MicheleUßmüller, ThomasDong, QianMagnussen, SteenUy, BrianDong, Changming Magri, LucaVacher, MichelDong, YanchaoMahabadi, Kambiz AnsariVaghefi, RezaDong, LiliMahadeva, Suresha K.Vai, Mang I.Dong, DamingMahgoub, MohamedVaishakh, ManuDong, XiaochenMahmoudian, NinaValcárcel, MiguelDong, BoMAINETTI, LUCAValente, JoãoDong, JunhangMaio, Francesco DiValera, Diego L.Dong, FengzhongMajdzik, PawełValerio, Valerio DiDonno, Dario Majji, ManoranjanValero, EnriqueDonzella, ValentinaMajumder, AditiValladares, Luis De Los SantosDorizzi, BernadetteMakeyev, OleksandrValova, IrenDormido-Canto, SebastianMAKHOUL, AbdallahVan, VienDornaika, FadiMaksymiuk, Krzysztofvan Bennekom, W.P.Dörner, RalfMalara, PietroVan Dam, MikeDorronzoro, EnriqueMaldonado-Basilio, RamónVan De Vijver, EllenDorsey, Kristen L.Maleki, SinaVan Den Abeele, FlorisDosen, StrahinjaMalkin, RobertVan Den Noorta, Josien C.Dou, XincunMalocha, Donald C.Van Der Zande, DimitryDovis, FabioMalumbres , Manuel Perez van Hees, VincentDragone, MauroMamun, QuaziVan Helden, J. H.Drap, PierreMan, ZhihongVan Hirtum, AnnemieDreesen, LaurentManaf, Asrulnizam Bin AbdVan Kleunen, WouterDrexler, PetrManamanni, NoureddineVan Laerhoven, KristofDrobizhev, MikhailMancini, AdrianoVan Ostayen, RonDrouin, BrianMandava, Ajay KumarVan Schoor, GeorgeDry, IanMandow, AnthonyVan Someren, MaartenDrysdale, TimManeuski, DimaVande Hey, JoshuaDu, KeManfredi, ClaudiaVanderelst, DieterDu, XiaosongMangiarotti, SylvainVanello, NicolaDu, BoManh Nguyen, CuongVanemon, JeanetteDuan, FajieManivannan, NadarajahVanlanduit, SteveDuan, Zhan-shengMankodiya, KunalVardasca, RicardoDuan, FabingMann, Danny D.Vargas, FranciscoDuan, DewenManolopoulos, SpyrosVargas-Rodriguez, EverardoDuann, Jeng-Ren (JR)Mansinha, LaluVarotsos, CostasDubey, AshishMansoor, RiyadhVasilakos, AthanasiosDuerkop, AxelMantri, DnyaneshwarVasseur, PascalDufour, SuzieMantzaris, DimitriosVatsalan, DinushaDunbar, Alan D.F.Manzin, AlessandraVaudenay, SergeDunbar, AlanManzino, Ambrogio M.Vázquez, DavidDuraia, El-shazly M.Mao, LucaVazquez Garcia, CarmenDurante, MartinMao, Zong-WanVazquez-Castro, ÁngelesDurduran, TurgutMao, DongVega, ShimonDurillo, Juan J.Mao, HanbinVehkaoja, AnttiDuro, RichardMao, HongjuVelotta, RaffaeleDurso, GuidoMarais, JulietteVeltink, Peter H.Duta, NicolaeMaran, FlavioVenkatesh, Y. VenkatakrishnaiyaDuval, MichelMarço, Paulo HenriqueVenticinque, SalvatoreDzantiev, BorisMarcos, JorgeVera, LA FERRARADziubla, ThomasMargui, EvaVerardo, RobertoDZIUDA, ŁukaszMariani, StefanoVerdoliva, L.Eaton, Mark JonathanMarie, RodolpheVerhagen, SandraEaton, BrettMarinakis, VangelisVerhoeven, GeertEconomou, AnastasiosMarinesco, StéphaneVerikas, AntanasEdgar, Thomas F.Marino, ArmandoVerikoukis, ChristosEdirisinghe, RuwiniMarjovi, AliVernier, PaoloEdiriweera, SisiraMarkelin, LauriVerykios, VassiliosEdoardo, SabbioniMarques, M. Joaquim BastosVette, AlbertEdwan, EzzaldeenMarquez Barja, JohannVettore, AntonioEfatmaneshnik, MahmoudMarrazza, GiovannaVezzetti, EnricoEgami, YasuhiroMarrone, StefanoVía, JavierEggeling, LotharMarsal, Lluis F.Viegas, DianaEhrhardt, DietmarMarsden, JonathanViikari, VilleEhtezazi, TourajMartalò, MarcoViitala, TapaniEielsen, ArnfinnMartalo, M.Vilas-Boas, joãoEisele, HeribertMartic, SanelaVilkko, MattiEjaz, WaleedMartin, AngelVilla, FedericaEl Abed, Abdel I.Martín, DavidVillacorta, Juan J.El-Darymli, KhalidMartin-Gorriz, BernardoVilladangos, JesusEl-Omari, SamirMartin-Lopez, SoniaVillani, Maria GabriellaEl-Osery, Aly I.Martínez, JoaquínVillarrubia, GabrielEl-Safty, Sherif A.Martínez, José F.Vinai, RaffaeleElarabi, TarekMartinez, ManuelVinel, AlexeyEliasson, JensMartínez Torres, JavierVinuesa, Xabier AnguloElishakoff, IsaacMartínez-Ballesté, AntoniViricelle, J.-P.Ellis, JonthanMartinez-Garcia, EdgarVisser, ArnoudElmahgoub, KhaledMartinez-Manez, RamonVisser, FleurElmasry, GamalMartinez-Perdiguero, JosuVitetta, Giorgio MatteoElRahman, AmrMartinho, EditeVittucc, CristinaEmadi, Tahereh ArezooMartins, RodrigoVivas Albán, Oscar AndrésEmardson, RagneMartins, HugoVivier, VincentEmeis, StefanMartins-Filho, Luiz S.Vlassov, SergeiEmokpae, LloydMartinsen, Ørjan G.Vo, Ba-NguEncarnação, PedroMartyniuk, MariuszVohland, MichaelEndo, TatsuroMas, IgnacioVoiculescu, IoanaEngelen, Peter-JanMasani, KeiVolckaert, BrunoEngstrand, ChristinaMashimbye, EricVollmer, FrankEntezami, ManiMasiello, GuidoVolmer, MariusEpifani, MauroMasiero, AndreaVolz, JürgenErathodiyil, NandananMaskulk, MdVona, MarsetteErdem, EmreMassa, A.Vong, Chi-ManEriksen, René LyngeMassucco, StefanoVoss, Kenneth J.Ernst, SvenMasuda, RyosukeVosselman, GeorgeErtl, PeterMatagne, ErnestVu-Van, HiepEsaki, HiroshiMatellán Olivera, VicenteVuolo, FrancescoEscalona, O JMaterer, Nicholas F.Vyskočil, VlastimilEscot-Bocanegra, DavidMatese, AlessandroWachowiak, AndreEsfandyarpour, RahimMathiopoulos, P. TakisWada, AtsushiEslamian, MortezaMatias, Ignacio RaulWagner, WolfgangEspinoza, Pedro MagañaMatko, VojkoWagner, BernardoEsquerre, CarlosMatos, AníbalWagner, Stefan RahrEuser, TijmenMatsko, AndreyWagner, ThorstenEvans, CainMatson, Eric T.Wahab, M.A.Everett, MarkMatsumoto, MitsuharuWakamiya, Prof.EVTUGYN, GennadyMatsumoto, SatoshiWakui, ShinjiExell, TimMatsumura, KentaWalawender, Jakub P.F. Ochoa, SergioMatsuya, IwaoWalczak, RafałFabio, VianelloMatta, EricaWalczak, KrzysztofFabregas, ErnestoMatteo, Andrea DiWalczykowski, PiotrFacchinetti, TullioMattetti, MicheleWallace, LukeFadel, EtimadMattiasson, BoWalsh, Kevin M.Faezipour, MiadMavrikios, DimitrisWalter, UlrichFalasconi, M.Maybank, SteveWan, JiafuFalchi, FabioMayer, ChristopherWan, Kai TaiFalco, GianlucaMayer, AndreasWan, YanFalcone, FranciscoMayer, DirkWang, Chung-hoFalkman, GoranMazurek, PrzemysławWang, YunjiaFallavollita, PascalMazza, ClaudiaWang, HongqiangFalletti, EmanuelaMazzotti, MatteoWang, PingFan, XinxinMcalpine, MichaelWang, Sheng-ShihFan, ShoushanMcCaig, Heather C.wang, junboFan, GuoliangMcCarthy, JohnWang, AiminFan, NengMcCullagh, PaulWang, YujieFan, XudongMcDonald, Timothy P.Wang, ZhiFan, ShaoweiMcdonough, SuzanneWang, GuoquanFang, XiaoshengMcEwan, AlistairWang, JunFang, YeMcGibney, AlanWang, WeiFang, HongliangMcGinley, BrianWang, ZenengFang, Shih-HauMcGinnis, Ryan S.Wang, Chi-YoungFang, JianchengMcilroy, David N.Wang, XiaogongFang, YumingMcKeever, SusanWang, LeiFang, GengfaMcLamore, EricWang, YunhongFang, PengMcManus, Barry J.Wang, YunFangjiong, ChenMcmillin, Bruce M.Wang, YuanpengFantner, GeorgMcMurrough, C. D.Wang, YijunFantoni, RobertaMcnamara, ShamusWang, ChengyuanFantozzi, SilviaMcshane, MichaelWang, JianfangFarha, OmarMcWilliam, StewartWang, JingchuanFariborz, EntezamiMedeiros Ferreira, João P.Wang, ZhenxinFarr, ThomasMedvidović-Kosanović, M.Wang, HeshengFarshad, ArshamMeffin, HamishWang, HaiFathi, HabibMehta, PrashantWang, WenFaurholt-Jepsen, MariaMeinhardt-Llopis, EnricWang, Kevin I.-K.Fay, Patrick J.Melero-Martin, Juan M.Wang, JieFazenda, BrunoMelia, UmbertoWang, DongFazio, Albino LemboMelicio, RuiWang, HongbingFeber, Boris LeMellbye, Brett L.Wang, JunyuFegadolli, William S.Mellone, SabatoWang, YanFeger, ReinhardMelo-Pinto, PedroWang, ChaoFeichtinger, Hans G.Meloni, AlessioWang, KeminFeintuch, AkivaMemarovic, NemanjaWang, JianguoFelici-Castell, SantiagoMen, HongWang, YingFelisberto, PauloMendão, RicardoWang, ShaopengFeng, LishuangMendes, Paulo MateusWang, Jian-nengFeng, Zhi HuaMéndez, A.Wang, HongmeiFeng, Maria Q.Menegaldo, Luciano L.Wang, David ZhiweiFeng, ShaojunMenelas, Bob-AntoineWang, JigangFeng, ZhipengMenezes, PauloWang, Tie-JunFensli, RuneMeng, GuowenWang, X.Fenstermaker, LynnMeng, QinghaoWang, XiaofengFentanes, Jaime PulidoMenges, FabianWang, Yao-tienFerapontova, Elena E.Menna, FabioWang, YongFerentinos , Konstantinos P. Mercorelli, PaoloWang, DanweiFerguson-Pell, MartinMerino, LuisWang, Dian HuiFernandes, ElisabeteMerlo, SabinaWang, GuijinFernandes, Ricardo JorgeMerola, FabienneWang, MeiqinFernández, RoemiMerschrod, ErikaWang, LeyuFernández, Jesús CapitánMesas-Carrascosa, F. J.Wang, HuihuiFernández Caballero, AntonioMescheder, UlrichWang, ZinanFernández Melián, SuselMeschino, SimoneWang, Yu-LinFernández Sarría, AlfonsoMessori, LuigiWang, YunshengFernández-Blanco, EnriqueMetsis, VangelisWang, Alan X.Fernández-Hernández, IgnacioMettler, Bérénice Wang, ZheFernando, AnuraMeulenberg, ElineWang, Xu-dongFernando, NuwanthaMeunier, JeanWang, Zizhong JohnFernando, Vidal-VerdúMeyer, Jerry R.Wang, KeFerraz, LuisMiao, ZhenweiWang, Gou-JenFerreira, MichelMiao, YinpingWang, JianFerreira, ElnatanMicangeli, AndreaWang, WenqinFerreira, João FilipeMichahelles, FlorianWang, HuaFerreira, Hugo A.Michal, CarlWang, BangFerri, GabrieleMichalak, MarcinWang, Jia-chingFerri, GiuseppeMichalska, AgataWang, Mu-chunFerruz, JoquínMichel, Carlos R.Wang, XingweiFfrancois, AlexandreMichelena, MarinaWang, YifanFicco, MassimoMichelson, DaveWang, NaigangFidan, BarisMigliaccio, MaurizioWang, YaojinFidanova, StefkaMigliorato, MaxWang , Liangmin Figorito, BenedettoMiguel, Gonzalo DeWang, HaiboFigueira, RitaMiguel-Bilbao, Silvia DeWang, TanyuFigueiredo, LinoMihaylova, LyudmilaWang, Chua-ChinFilatrella, GiovanniMikada, HitoshiWang, RuhaiFilipe, Doutor Vitor Manuel JesusMikhailova, ElenaWang, LiqiangFilipe Costa, ManuelMikhelson, Ilya V.Wang, Tsan-pinFilippi, AnthonyMiki, HirofumiWang, Ming-ShyanFilomena Camões, MariaMiki, NorihisaWang, Chia-PinFingas, MervMikita, TomášWang, RenzhongFinocchio, GiovanniMikoshiba, KatsuhikoWang, BinbinFiorani, LucaMikropoulos, Tassos A.Wang, Chau-changFiorini, CarloMikš, AntonínWang, HaikouFischer, AndreasMilanés, VicenteWang, Faming (Felix)Fischerauer, GerhardMilanova, FaniWang, QiuhuaFlammini, FrancescoMilej, DanielWang, ZengcaiFlater, Erin E.Milella, AnnalisaWang, ChengFlechsig, Gerd-uweMiller, PhillipWang, XiqinFleming, KevinMiller, JimmieWang, Chi-KueiFlower, StephenMiller, PaulineWang, ZhaohuiFofana, IssoufMin, JunhongWang, QunmingFong, SimonMinakuchi, ShuWang, Li (Lily)Fong, LouisMineno, HiroshiWard, MichaelFonollosa, JordiMing, HanWard, LeighFonollosa, J.Miribel, Pedro LuisWard, PhilFonseca, Viviane MunizMiron, SebastianWard, Jonathan M.Fonseca, Teresa de Jesus FidalgoMiroshnichenk, Andrey E.Warren, MichaelFontecha, JesúsMirov, SergeyWarwick, MichaelFoo, ErnestMirsky, Vladimir M.Wasicek, ArminForesti, LorisMisgeld, BernoWasisto, Hutomo SuryoForster, Carlos Henrique QuartucciMisic, JelenaWatanabe, EikiFort, AdaMiskowicz, MarekWatanabe, TetsuyouFortino, GiancarloMisron, NorhisamWaterlot, ChristopheFortuna, LuigiMitchell, JohnWatkins, Anthony NForzani, EricaMitchell, ChrisWatts, DoyleFoschini, LucaMitronova, GyuzelWatzenig, DanielFOSSATI, Caroline VèveMitsugi, JinWeber, Rolf H.Fotiadis, DimitriosMitton, NathalieWeber, RobertFotopoulos, GeorgiaMiura, JunWebster, JohnFotouhi, HosseinMiyake, ShiroWebster, SarahFouchal, HaceneMizsei, JánosWeeber, Jean-ClaudeFoucher, SamuelMizuno, HideakiWei, Shun-JunFouhey, DavidMizutani, YoshihiroWei, JianmingFourati, HassenMłyniec, AWei, Chia-HungFourati, NajlaMo, HainingWei, Leifournier-viger, philippeMöbus, ClausWei, YangFox, GlenModin, OskarWei, XiukunFox, Neil I.Modotto, DanieleWei, Chia-PoFraga, M. A.Moeslund, ThomasWeidner, A.Franceschinis, MirkoMoh, SangmanWeigand, AnnikaFrancesco, ChiavaioliMohan, Ananda S.Weinberg, MarcFrancesco, BianconiMohd-Yasin, FaisalWeinmann, MartinFrancioso, L.Mohri, KaneoWeiss, JasonFrancis, DanielMolina, JavierWelch, KenFrancisco Javier, Cardenal EscarcenaMolina, Ana IsabelWen, Chih-YuFranco, RicardoMolina Cantero, Francisco J.Wen, Tzai-Hung Franco, AnnalisaMolinari, FilippoWen, FuxiFrasconi, ChristianMolnár, GáborWendt, KlausFraternali, FernandoMomayez, MoeWenger, ErichFratoddi, IlariaMonaghan, DavidWerner, MartinFreddi, AlessandroMönch, IngolfWestafer, Ryan S.Freestone, DeanMondal, DebashisWetterlind, JohannaFregonese, LuigiMondal, Md. SurabuddinWeyn, MaartenFreire, DavidMongelli, MaurizioWhalley, RichardFrenken, MelinaMonico, João F. G.Wheeler, NatalieFriedt, Jean-MichelMonkawa, AkiraWhelan, ThomasFrischholz, RobertMönnigmann, MartinWhite, RyanFritzen, FelixMonserrat, OriolWhitehead, AnthonyFritzsche, MarcoMonteiro Santos, FernandoWhitehead, KenFrizera, AnselmoMontesarchio, DanielaWhiteside, TimFrontoni, EmanueleMontez, CarlosWhitty, Mark AlbertFrosch, TorstenMontoliu, RaulWieczorek, PiotrFu, L.L.Montoya, AngelWielgosz, PawelFu, ShengMonzón-Hernández, DavidWiersig, JanFu, HuirongMoore, PaulWieser, ManfredFu, YuningMoore, PhilipWietfeld, ChristianFuentes-Fernández, RubénMoore, Sean D.Wijkstra, H.Fuh, Chiou-shannMora, AndreWild, PeterFujita, TakayukiMorais, Jorge E.Wilder-Smith, PetraFukuda, TakeshiMorales, S.wilkens, volkerFung, Wai-keungMorandi, LucaWilkinson, MaxwellFurferi, RoccoMoranduzzo, ThomasWillander, MagnusFusiek, G.Moreau, MarcWillers, JeffreyFusiello, AndreaMorell, AntoniWilliams, BillyFutagawa, MasatoMoreno, FélixWilliams, TrishGabriel, Jose luisMoreno, LuisWilson, BenjaminGadella, Theodorus WJMoretto, Ligia MariaWilson, David F.Gagar, DanielMorgan, SteveWilson, Alphus D. Gage, Joann R.Morganti, P.Wischniewski, SaschaGaglione, SalvatoreMorgenthal, GuidoWismüller, RolandGajic, DragoljubMorillo, CarlosWithayachumnankul, WithawatGalán-Mascarós, José RamónMorimoto, YasuhikoWitte, Luc P. deGalan-Vidal, Carlos AndresMorioka, KazuyukiWitte, HartmutGalante, AngeloMoriondo, MarcoWojciechowski, AdamGalezowska, JoannaMoron, CarlosWolbrecht, EricGall, JuergenMorris, BrendanWolf, Marilyn CGalland, FredericMorsi, YosryWon, Jong-HoonGallego, JuanMortazavi, Jack BobakWon, Chee-SunGalstyan, VardanMoschitta, AntonioWon, Chang-HeeGalván-Tejada, Carlos E.Moschner, Christian R.Wong, Tak SingGalve, RogerMoscone, DanilaWong, LawrenceGambi, EnnioMoshou, DimitriosWong, Yiu-chungGambs, SébastienMoshtaghi, MasudWong, Pak-kinGamo, JavierMostofi, YasaminWong, KainamGams, AndrejMotai, YuichiWong, Rex H.Gams, MatjažMotta, Gian MarioWood, BaydenGan, NingMouchtaris, AthanasiosWood, TroyGandino, FilippoMoustafa, AhmedWoodford, BrendonGao, XiaofengMoy, MatthieuWright, BillGao, WeiMoya, FranciscoWu, XiangyangGao, David (Zhiwei)Mudanyali, OnurWu, QuincyGao, ZhiqiangMujica, Luis EduardoWu, Wei-chiangGarcía, FernandoMukesh Kumar, Mukesh K.Wu, HongyiGarcia, NicolasMukherjee, Partha SarathiWu, Shin-TsonGarcía, José M.Mukhopadhyay, Subhas C.Wu, Yik-ChungGarcía, RobertoMüller, GeorgWu, DaleiGarcia, Javier RoseroMüller, RichardWu, Chao-chengGarcia, JesusMuller, CobusWu, XiaomingGarcia de Jalon, JavierMunafo, AndreaWu, XiaoshengGarcía Mateos, GINÉSMunge, BernardWu, Hai-longGarcía Rodríguez, Carmelo RubénMunilla, JorgeWu, XiongbinGarcia Zapirain, BegoñaMunir, TajWu, XinjunGarcía-Lamont, FaridMunoz-Gama, JorgeWu, ZhengGarcia-Morchon, OscarMuñoz-Gea, Juan PedroWu, RuihengGarcía-Orellana, Carlos-javierMünzenrieder, Niko S.Wu, GuoyuanGarcía-Otero, MarianoMura, AndreaWu, ChuandeGarcia-Sanchez, Antonio-JavierMurao, KazuyaWu, Shu-PaoGarcia-Sanchez, FelipeMurayama, RiichiWu, XiaolinGarcia-Santos, VicenteMurimi, RenitaWu, QiGarcia-Teodoro, PedroMurino, VittorioWu, GuofengGargiulo, Gaetano DMuroyama, MasanoriWu, LifengGarozzo, DomenicoMurphy, AndrewWu, ZhiluGarratt, EliasMurray, IainWu, ChenshuGarrido, AngelMusić, JosipWu, Jianhua (jerry)Garzon, MarioMussetta, MarcoWu, MuqingGarzon, E. M.Mustafa, Mohamad Y.Wu, YuanxinGasparrini, SamueleMusumeci, LucianoWu, WeiGasti, PaoloMyers, Oliver J.Wu, JKGazda, JurajMyers, ChrisWu, ZhenyangGazerani, ParisaMyers, MikeWu, XiaopingGe, MaorongMyung, HyunWu, Yue IvanGe, XinNafarieh, AlirezaWu, Ren-JangGea, MiguelNagamatsu, TakashiWu, YuanmingGebhardt-Henrich, Sabine G.Nagao, TadaakiWu, XiaojunGebre, Biruk A.Nagatani, KeijiWu, NanGeddis, Demetris L.Nagel, Jacquelyn K.S.Wu, MingquanGeernaert, ThomasNaghdy, FazelWu, Jui-yuGeissinger, PeterNaik, GaneshWu, XiaobeiGelembe, ErolNajar, HadiWu, JianfengGemeiner, PeterNakaji, TatsuroWunderlich, Ch.Gemert, Jan vanNakamura, TomoyaWünsche, BurkhardGenest, MarcNakano, KimihikoWymeersch, HenkGeng, JianghuiNakano, MichihikoWynter, LauraGeng, YunhaiNakano, TadashiWysocki, LauraGeorge, SuciuNakata, MakikoWysocki, GerardGeorgieva, ElenaNakayama, MasaharuXhakoni, AdiGeorgopoulos, AndreasNakazawa, AtsushiXia, LinyuanGeorgoulas, ChristosNakhostin, M.Xia, DunzhuGeorgoulas, GeorgeNalayanda, DivyaXia, YuanqingGerdon, Aren E.Nam, YoonkeyXia, Ke YuGerhard, DavidNamour, Ph.Xia, LuGerke, MarkusNamuduri, KameshXia, YongGernaey, Krist V.Nanayakkara, ThrishanthaXia, ChunleiGerosa, RodrigoNandy, Anil KumarXian, YuezhongGhanbarpour, HabibNanni, LorisXiang, TaoGhio, AlessandroNaraghi-Pour, MortXiangjun, XinGhisi, AldoNaranjo Hernández, E.Xiao, YangGhorayeb, Sleiman R.Nardi, DamianoXiao, ZhifengGhorpade, AjinkyaNarieda, ShusukeXiao, QinghanGhosh, PreetamNasr, BabakXiao, JohnyGhosh, RajatNasridinov, AzizXIAO, JiangGiacobbe, MaurizioNasrollahi, KamalXiao, BoqiGiacomozzi, ClaudiaNassar, SamehXiao, ZhengguoGiani, AnnaritaNassiopoulou, AndroulaXie, GaogangGiannazzo, F.Nati, MicheleXie, ZhijianGiantsoudi, DrosoulaNavarro, Jorge A. R.Xie, HuiGibaldi, AgostinoNavarro, Antoni PérezXing, ZishengGibbens, PeterNavarro-Pedreño, JoseXiong, JipingGibson, DesNavas, JesusXiong, JijunGidlund, MikaelNawaz, SarfrazXiong, ShupingGikas, VassilisNawrat, AleksanderXiong, HaoyiGil, EmilioNazirizadeh, YousefXiong, FeiGilestro, Giorgio F.Ndieyira, JosephXu, FeiGill, RonNeasham, JeffXu, YongGillespie, AlanNebot Roglá, PatricioXu, LiangGiordano, DomenicoNee, A.Y.C.Xu, GuobaoGiorgetti, AndreaNef, TobiasXu, JiangtaoGiorgi, GabrieleNefti-Meziani, SamiaXu, YongjunGiorgio, QuerNehorai, AryeXu, XinGiouroudi, IoannaNeitzel, FrankXu, YanGirault, HubertNelson, CarlXu, XinlongGiudice, Nicholas A.Nemova, GalinaXu, SenGiuliani, RobertaNener, BrettXu, LongxiaGiuseppe, QueroNeri, GiovanniXu, ChuanglongGjevestad, Jon GlennNerino, RobertoXu, ChongbinGlass, John D.Neska, AnneXu, ChunyeGleich, DusanNeugebauer, UteXU, JingGlennie, CraigNewe, ThomasXu, LinaGlentis, George-OthonNewlands, Nathaniel K.Xu, LanGlisic, BrankoNewman, Kimberly E.Xu, ZhaochaoGlover, Ian A.Newman, RobertXu, XiangguoGodfrey, AlanNewman, Timothy S.Xu, WeiheGoes Oliveira, JorgeNex, FrancescoXu, HuirongGokaraju, BalakrishnaNg, KcXu, BaoguoGoldberg, LynetteNg, Wai TungXu, WanliGolen, Erik F.Ng, T. W.Xu, RichardGollmann, DieterNg, JasonXu, XunGoltz, DouglasNg, AndyXuan, Fu-zhenGómez, Ernesto SuasteNgoko, YanikXuan, XiangchunGómez Mancilla, Julio CésarNguyen, CuongXue, NingGomez-Gonzalez, M.Nguyen, Linh VietXue, WeiGonçalves, RicardoNguyen, HoangXue, BindangGoncalves, LuisNguyen, Nam-TrungXue, XinyuGong, XiaojinNguyen, HungXue, KaipingGong, JiaqiNguyen, T. HienXue, Zi-LingGong, Myoung-seonNguyen, LinhYacoot, AndrewGong, QiumingNguyen-Thanh, Dr. NhanYafa, LevanonGonzále, RamónNi, BingbingYakes, BetsyGonzalez, MiguelNichols, AndrewYamamura , MasakiGonzalez, JoaquinNiedźwiecka, AnnaYamato, MasayukiGonzalez, Luis Sanchez Nielsen, Michael MeinildYan, RuqiangGonzález, Antonio GuerreroNielsen, ShawnYan, GongminGonzález Aguilera, DiegoNiese, FrankYan, BoGonzález-Piqueras, JoséNieto, MarcosYan, Wai YeungGonzalo Garcia, CerruelaNikolic, JanoschYan, JizeGoossen, KeithNiku, SaeedYang, Jinn-MinGopalakrishnan, BhaskaranNilsen, Wendy J.Yang, LingGordon, DawudNilsson, ErikYang, Chuan-kaiGordon, Timothy J.Nilsson, Jan TobiasYang, NingGorricho, Juan-LuisNing, HuanshengYang, LanGoryll, M.Ning, XiaolinYang, Chang-BiauGosalbez, JorgeNirjon, ShahriarYang, Lian-xiangGoshtasby, A. ArdeshirNitti, MicheleYang, ChungangGoswami, SantonuNiu, YugangYang, ZhenchuanGotchev, AtanasNiu, HeYang, ShikuanGottschlich, CarstenNocerino, EricaYang, BishengGoudarzi, NavidNoguer, ThierryYang, SungGouy-Pailler, CédricNoh, YohanYang, Y.JGoverdovsky, ValentinNoland, Thomas L.Yang, XiaodongGowans, EricNolesini, TeresaYang, BoGozdowski, DariuszNoras, MaciejYang, Bobby Mee LoongGracia-Espino, EduardoNordblad, PerYang, YixinGrana, CostantinoNordmeyer, HenningYang, Lung-JiehGranade, Timothy C.NOSHADI, AminYang, JinfengGranjal, JorgeNoubir, GuevaraYang, JianyiGrant, Olga M.Novak, DomenYang, RusenGrant, JamesNovak, RichardYang, MoGrasso, FrancescoNowak, RobertYang, RuigangGrate, Jay W.Nowicka, Anna Maria Yang, S.Y.Grau, AntoniNowotny, ThomasYang, GengGravina, RaffaeleNüchter, AndreasYang, ShuhuiGreenbaum, AlonNuño, FernandoYang, Ming-DerGreener, JesseNunziata, FerdinandoYang, YutingGrega, MichałNurmi, JariYang, WenmingGregory, Mark A.Nuske, StephenYang, MingleiGreiner, DavidNwankire, Charles E.Yang, SamGrejner-Brzezinska, DorotaNyka, KrzysztofYang, PoGreve, DavidO'callaghan, DonalYang, Ming-JenGriggio, FlavioO'Driscoll, CillianYang, Chin-LungGrill, RomanO'young, SiuYang, Yee-PienGrimm, PaulO’Mahony, ConorYang, Yu-MingGross, Jason N.Oancea, BogdanYang, YingchenGrosse, Christian U.Oddo, CalogeroYang, WuqiangGroves, RogerOdeh, Suhail M.Yang, DingqiGruber, JonasOdijk, DennisYang, Eileen Chih-YingGruev, ViktorOeffinger, Donna J.Yang, JiachenGrulkowski, IreneuszOesterschulze, E.Yang, ShumingGrunwaldt, Jan-DierkOguz, Huseyin KaganYang, ZheGruyer, DominiqueOh, HyondongYang, Chan-suGrzebieta, RaphaelOh, Yu-kyoungYang, TangwenGu, YuOh, Min-cheolYang, Hee-DeokGu, FuboOh, Sang-HoYang, MingGu, QuanquanÖhberg, FredrikYang, HaigangGu, Bon-GwanOhodnicki, Paul R.Yanovsky, IgorGU, FuqiangOhtsu, MasayasuYao, NianminGualandi, IsaccoOkamoto, Y.Yao, JasonGuan, HaiyanOkarma, KrzysztofYao, TianGuan, ZhangyuOkawai, HiroakiYao, Wu-SungGuan, YanOkazaki, ToshiyaYao, JiafengGuarino, S.Okuma, MasaakiYao, Chun-WeiGuarnera, Giuseppe ClaudioOKUMURA, SusumuYao, SuyingGubbi, JayavardhanaOlcum, SelimYarbrough, JohnGuedes, LuizOldham, KennYaroslavsky, Leonid P.GÜEMES, ALFREDOOleinick, AlexanderYe, MaoGuérard, JeanOletic, DinkoYe, BaoxianGuerrero, CesarOlivares-Mendez, Miguel A.Ye, XuesongGuerrero, JosechuOlive, M. FosterYeh, Sheng-ChengGuerrier, StéphaneOliveira, Sérgio CampelloYeh, Wei-ChangGuerrieri, AntonioOliveira, João P.Yelamarthi, KumarGuerriero, FrancescaOliveira, Elcio C.Yen, C.W.Guido, GiuseppeOliveira, Hélder F. P. DeYeom, SeokwonGuijt, Rosanne M.Oliver-Codina, GabrielYerushalmi, LalehGuillaume, DUCARDOlivier, Le TraonYi, JiabaoGuillon, QuentinOlivier, PhilippeYi, TinghuaGuimaraes, Dayan AdionelOlmi, GiorgioYi, JingangGuinaldo, MaríaOlson, Michael W.Yilmazer, NuriGuitton, AlexandreOlthuis, W.Yin, WuliangGuivant, JoseOlyaee, SaeedYin, YuehongGuldiken, RasimOmbres, LucianoYing, LimingGulinatti, AngeloOmholt Gjevestad, Jon GlennYingling, Yaroslava G.Gullström, MartinOnen, OnursalYiping, WangGun’ko, YuriiOneto, LucaYlianttila, MikaGunawidjaja, RayOng, Eng-JonYoe, HyunGunningberg, PerOnieva, EnriqueYokoyama, ShiyoshiGuo, Amy WeihongOno, KanjiYokoyama, YoshihitoGuo, ShiruiOnoe, MorioYomsi, Patrick MeumeuGuo, GuodongOpitz, MadeleineYoo, Kee-youngGuo, YanhuiOrazi, LeonardoYoo, SeehwanGuo, DiOrdóñez, Fco. JavierYoo, Seong-eunGuo, FeiOrdoñez, CelestinoYoo, ByungseokGuo, BinOrganero, Mario MuñozYoo, HongseokGuo, BaofengOrmond, OlgaYoon, HargsoonGuo, FuchunOrsini, AndreaYoon, SeokhoonGurkan, Umut AtakanOrtega, Francisco R.Yoon, SeongkyuGururajan, SrikanthOrtiz, AlbertoYoon, Sung-GukGustafsson, PeikOrtiz, FranciscoYoon, Yong-KyuGutiérrez, Juan ManuelOspina, GustavoYoon, GilwonGutierrez-Blanco, OscarOstasevicius, VytautasYoon, Yong-IkGutiérrez-Capitán, ManuelOstendorf, BertramYoon, MinjoongGuy, ChrisOstermann, Frank O.Yoon, JuyoungHaala, NorbertOstrowska, KseniaYoon, YongjinHaas, RüdigerOsuch, TomaszYoshida, SatoshiHabib, AymanOszust, MariuszYoshimoto, ShusukeHabisreuther, TobiasOteiza, IgnacioYoshimoto, ShigekaHachem, SaraOtnes, RoaldYoshino, KenjiHaddadi, KamelOtsuka, YuichiYoshinobu, TatsuoHadjiefthymiades, StathesOu, ShumaoYoshitomi, YasunariHaenen, KenOu, JianzhenYou, ZhengHaick, HossamOwens, Casey M.Younis, MohamedHalder, SebastianOyekan, JohnYousaf, AdnanHale, W.MicahOzturk, YusufYu, Shui YuHalfield, JerryPacheco, João G.Yu, YuebinHall, Drew A.Paciornik, SidneyYu, FeiHall-Beyer, MrykaPaczkowski , SebastianYu, HongyuHämmerle, MartinPadhye, NikhilYu, MingHammoudeh, MohammadPadilla-Castaneda, M. A.Yu, DongxiaoHampai, DariushPadua, LucaYu, YunyueHan, Jen YuPaeng, Dong GukYu, Chin-pingHan, ArumPage, WyattYu, JinHan, MinPaik, JoonkiYu, LuoHan, LujiaPaixão, Thiago R.l.c.Yu, FabiaoHan, JungongPal, SudeshnaYu, HuiminHan, DaewooPala, NezihYu, GuoHan, B.g.Palacin, JordiYu, GangHan, Il SongPalacios, AntonioYuan, JunsongHan, Jae-hungPalecek, EmilYuan, QiangQiangHan, Dong YeobPalleschi, VincenzoYuan, WeizhengHan, SeungwooPalli, GianlucaYuan, BinHan, Young-geunPalma, Alberto J. Yuan, ZhenHan, SangUkPalmer, JeffreyYuan, LeiHan, GunheePalomares-Salas, José CarlosYuan, QilongHan, YoukyungPalzer, StefanYuan, FeiHanaor, DorianPan, BingYuan, ShenfangHancke, GerhardPan, FeifeiYue, JianHang, Hsueh-MingPan, ChenYun, JaeseokHanke, KlausPan, Meng-shiuanYun, Kwang-SeokHanlen, LeifPan, FengYusa, NoritakaHanna, RadeckaPan, M.-CH.Yusa, Shin-IchiHänsch, RonnyPan, CaofengZabini, FlavioHao, JiangPan, ZhongyongZabulis, XenophonHara, MotoakiPan, XiangZamansky, RemiHara, ShinsukePandarese, GiuseppeZamorano, JaimeHaring, ChristianPandey, GauravZanardi, ChiaraHarith, M. A.Pandey, GunjanZanella, AndreaHarkonen, JaakkoPanfeng, HuangZapata, FelipeHarle, RobertPankanti, SharathZapién, Hiram GaleanaHarnefors, LennartPankratius, VictorZapotoczny, PiotrHart, John P.Panneton, BernardZappatore, MarcoHarvey, RichardPaoli, GeorgeZariffa, JoseHasager, Charlotte BayPaolo, BellavistaZarifi, Mohammad H.Hasan, AbbasPapa, MauricioZatar, WaelHasegawa, MasumiPapaelias, MayorkinosZayegh, AladinHashizume, HiromichiPapageorgiou, Xanthi S.Zelek, John S.Hashsham, Syed A.Papagiannakis, GeorgeZeltner, RichardHassan, QuaziPapaioannou, ThanasisZeng, RongHassan, ModarPapakiriakopoulos, DimitrisZeng, HulieHatzinakos, DimitriosPapoutsis, IoannisZeng, TieyongHauta-Kasari, MarkkuPappas, NikolaosZeng, WenHavlík, JanParajuli, SumanZeng, QinghuaHawes, MatthewPardalos, PanosZeni, LuigiHawrylak, Peter J.Paredes, JacoboZennaro, MarcoHayami, ShinyaParenthoen, MarcZeqiri, BajramHayashibara, YasuoPark, Myong-soonZhang, HaijunHayward, Jason P.Park, B. HyleZhang, XiaopengHazrati, MehrnazPark, Hyo SeonZhang, WenzengHe, TaoPark, ChungHyukZhang, XianfengHe, DavidPark, Yong-LaeZhang, YongjunHe, BinPark, Sang-HongZhang, TaoHe, JinliangPark, JaesikZhang, ZhaoxiangHe, JingPark, JungyulZhang, JianrongHe, YejunPark, Sung HaZhang, ShengpingHe, LiFengPark, SehyunZhang, YanpingHe, LinaPark, ChansikZhang, YinHe, HongshengPark, Won IlZhang, ZhiqiangHe, LiliPark, EdwardZhang, QijinHe, DongfengPark, Joon-ShikZhang, HaiboHe, YuqingPark, Young JuneZhang, ShujuanHe, ShiboPark, ByungwoonZhang, GuanglieHe, Yuanpark, NamjeZhang, JueHebert, PatricPark, Jae-HyoungZhang, ZhiguoHedayati, Mehdi KeshavarzPark, Kyi-hwanZhang, WeihuaHedley, John D.Parreu, Dra. IsabelZhang, RuiHeege, Hermann J.Parrot, SandrineZhang, YudongHeidari, HadiPartsch, UweZhang, WeipingHeidary, AliPasero, ErosZhang, DaqingHeinisch, MartinPasi, F.Zhang, BaochengHeinrich, S.M.Pasini, DarioZhang, LeiHeise, H.M.Pasolli, EdoardoZhang, HongHeise, Herbert MichaelPasquale, Fabrizio DiZhang, WenmingHeiskanen, ArtoPasqualetti, FabioZhang, ShanqingHelburn, RobinPasserone, RobertoZhang, YanHelder, DennisPassian, AliZhang, HouxiangHeldman, Dustin A.Pastoriza-Santos, IsabelZhang, MinweiHellen, EdwardPaterno, Aleksander SadeZhang, MingfengHemelrijck, Danny VanPaterson, MauraZhang, BowuHenault, Jean-MariePathan, Al Sakib KhanZhang, HaoHenderson, RitaPatrick, Christopher J.Zhang, YuHenebry, GeoffreyPatrono, LuigiZhang, ZhongpingHeneghan, ConorPau, GiovanniZhang, QianniHeng, LiangPaul, ManoranjanZhang, WeiHeng, Pheng AnnPaun, Maria-AlexandraZhang, QingHenricksen, MattPavlidis, George P.Zhang, YundongHensinger, W. K.Pavlík, ZbyšekZhang, XiaotongHer, Shiuh-chuanPavlovich, Christian P.Zhang, YunlinHernandez, NoeliaPawlowski, AndrzejZhang, ShanshanHernández, LuisPaya, LuisZhang, SongHernández-Pajares, ManuelPayne, Christopher JZhang, LongheHerranz, José Miguel Jiménez Paziewski, JacekZhang, YuanzhiHerrera-May, A. L.Pazos, AlejandroZhang, ZhenjiangHertlein, HeinzPearson, Matthew R.Zhang, ZhilinHervas, RamónPearton, S.J.Zhang, LihongHerzog, GrégoirePeci, Luis MiguelZhang, QichunHeunecke, OttoPecorella, TommasoZhang, RenyunHeydari, Shahram S.Pederson, ThomasZhang, FeihuHideshima, ShoPedro Santos, JoseZhang, JingJingHigashino, TeruoPeeters, BartZhang, HaihongHiggs, RussellPeham, ChristianZhao, YiHilkert, JamesPei, SongwenZhao, YanxiaoHill, RaymondPei, LingZhao, HongHillen, FlorianPejcic, BobbyZhao, XiangyongHinckley, StevenPeña-Arancibia, Jorge LuisZhao, XuefengHinds, G.Peñas, Matilde SantosZhao, ShuheHirakawa, KeigoPenezic, AbraZhao, LinHiromoto, R.E.Peng, YuchengZhao, ZhongliangHirose, KiyoshiPeng, Chien-fangZhao, YanHirsch, ThomasPeng, LihuiZhao, ShengkuiHirsch, E.Peng, JianZhao, WeishengHirtz, MichaelPeng, XiZhao, XiangweiHirvonen, LiisaPeng, ZhouhuaZhao, DongHmam, HatemPeng, ErwinZhao, JunHmimina, GabrielPenichet, VíctorZhao, FengHo, Chao-chingPenmatsa, VarunZhao , FengHo, Ho PuiPerales, Fco J.Zhao, JichaoHo, DerekPerallos, AsierZhao, PengHo, MichaelPerazzelli, PaoloZhao, FangHoath, StevePercoco, GianlucaZhao, YanjunHobiger, ThomasPerdigones, FranciscoZhao, JiangHodson, DavidPereira, EmilianoZhao, LiangHoffman, Anthony J.Pereira, RubemZhao, WeiHoffman, Matthew J.Pereira, Ernesto C.Zhao, XinHöflinger, FabianPerenzoni, MatteoZhen, LeiHol, Jeroen D.Peres, António ManuelZheng, YuanjinHolbein, WalterPeres, EmanuelZHENG, RenchengHölbl, MarkoPérez, José A. MorenoZheng, YuanqingHolgado, MiguelPerez, Rosazheng, shiqiangHoller, StephenPérez, Josefa LinaresZheng, CuieHolley, RachelPérez, JoshuéZherdev, Anatoly V.Holownicki, RichardPérez Oria, JuanZhong, Ray Y.Holzinger, AndreasPerez-Ruiz, ManuelZhou, HuiyuHong, MenPerez-Uribe, AndresZhou, JunHong, ZengPerhinschi, MarioZhou, Qian-YiHong, DaesikPeris-López, PedroZhou, FengfengHong, YoungdaePerles, AngelZhou, QifaHoogenboom, BartPeroulis, DimitriosZhou, GuoqingHooman, JozefPerrier, AurélieZhou, LiangHoorfar, MinaPersico, Adriano RosarioZhou, YuanxinHorgan, GrahamPersson, IngmarZhou, YongHori, MaiyaPeruzzi, AgneseZhou, ZhiHorng, Mong-fongPescaru, DanZhou, Xiao DongHorng, Shi-jinnPescosolido, LoretoZHOU, QiangHorobin, Richard WPestana, JesúsZhou, FugenHorrillo, María del CarmenPeteinatos, G. G.Zhou, ChangjianHorvath, GyörgyPeter, SteffenZhou, YufengHosokawa, RyujiPeter, HubinskýZhou, Ming-tuoHossain, A.K.M. AzadPetermann, KlausZhou, DengpanHossain, A.k.m. MahtabPetit, NicolasZhou, SusanHosseinnezhad, RezaPetit, JonathanZhou, Li-HuiHostettler, RolandPetr Skládal, PetrZhou, XunHottowy, PawełPetracca, MatteoZhou, DonghuaHou, ChengyuPetraglia, Mariane R.Zhu, GuizhiHou, Kun-meanPetroccia, RobertoZhu, YinianHoussineau, JeremiePetruškevičius, RaimondasZhu, JiHowey, DavidPettersen, ErikZhu, ChunHrad, JaromirPetti, DanielaZhu, Hong-HuHristoforou, EvangelosPfeiffer, MaxZhu, HongboHsia, Chih-HsienPfeiffer, HelgeZhu, YonggangHsiang, Tien-RueyPfiffner, FlurinZhu, LikunHsiao, Fu-yuenPfletschinger, StephanZhu, XinhuaHsiao, Rong-ShuePhan, Manh-huongZhu, DaqiHsiao, Shen-FuPhinyomark, AngkoonZhu, KunHsiao, Yung-ChiaPhothisonothai, MontriZhu, XiangyangHsieh, Chen-ChiungPiano, MartinaZhu, ZhigangHsieh, Ping-HsuanPicaud, SergeZhu, DibinHsieh, Shang-HsienPicerno, PietroZibordi, GiuseppeHsieh, Sheng-jenPieraccini, MassimilianoZihajehzade, ShaghayeghHsieh, Chih-ChengPierleoni, PaolaZijlstra, PeterHsieh, KuangwenPieters, RoelZilic, ZeljkoHsu, Miao-JuPietrzak, MariuszZimmerman, Patrick H.Hsu, Chih-shunPilcher, June J.Zimroz, RadoslawHsu, Shih-HsiangPiletsky, SergeyZingaretti, PrimoHsu, Chun-FeiPilny, L.Zitova, BarbaraHsu, Chien-changPilolli, RosaZondlo, MarkHsu, Ching-hsienPinahasi, YosiZou, XudongHsu, Yi-ChuPinchin, JamesZou, XiaoboHsu, Jin-chenPing, JianfengZou, QiongjingHsu, Heng-TungPinto, AlbertoZuba, MichaelHsu, WensyangPinto, Andry MaykolZude, ManuelaHu, NingPinto, AntónioZug, SebastianHu, JinlianPiotto, MassimoZulvia, Ferani E.Hu, JianjunPiras, AndreaZuo, XiaoleiHu, ZhongxuPIRO, BenoitZuperl, UrosHu, ManliPirozzi, SalvatoreZwiggelaar, ReyerHu, XiangyunPisano, RobertoZygielbaum, ArthurHu, Zhanyi



